# Isolation of Mixed Compositions of Cellulose Nanocrystals, Microcrystalline Cellulose, and Lignin Nanoparticles from Wood Pulps

**DOI:** 10.1021/acsomega.3c00295

**Published:** 2023-05-19

**Authors:** Tiffany Abitbol, Mikaela Kubat, Elisabet Brännvall, Nikolay Kotov, C. Magnus Johnson, Rustem Nizamov, Mikael Nyberg, Kati Miettunen, Niklas Nordgren, Jasna S. Stevanic, Maria Pita Guerreiro

**Affiliations:** † Institute of Materials, School of Engineering, EPFL, 1015 Lausanne, Switzerland; ‡ Bioeconomy and Health, RISE Research Institutes of Sweden, SE-114 28 Stockholm, Sweden; § Department of Chemistry, KTH Royal Institute of Technology, SE-100 44 Stockholm, Sweden; ∥ Department of Mechanical and Materials Engineering, Faculty of Technology, University of Turku, FI-20014 Turku, Finland

## Abstract

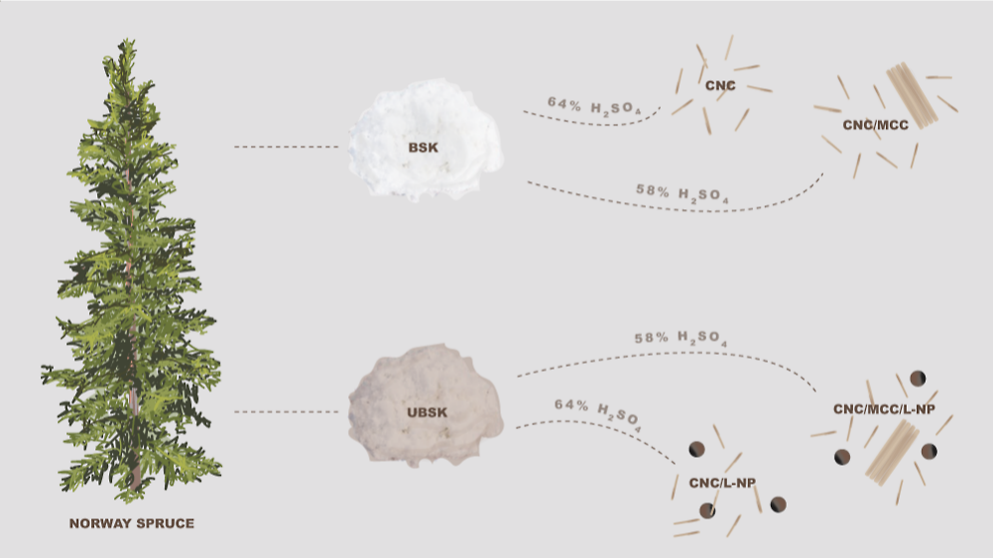

From a circular economy perspective, one-pot strategies for the isolation of cellulose nanomaterials at a high yield and with multifunctional properties are attractive. Here, the effects of lignin content (bleached vs unbleached softwood kraft pulp) and sulfuric acid concentration on the properties of crystalline lignocellulose isolates and their films are explored. Hydrolysis at 58 wt % sulfuric acid resulted in both cellulose nanocrystals (CNCs) and microcrystalline cellulose at a relatively high yield (>55%), whereas hydrolysis at 64 wt % gave CNCs at a lower yield (<20%). CNCs from 58 wt % hydrolysis were more polydisperse and had a higher average aspect ratio (1.5–2×), a lower surface charge (2×), and a higher shear viscosity (100–1000×). Hydrolysis of unbleached pulp additionally yielded spherical nanoparticles (NPs) that were <50 nm in diameter and identified as lignin by nanoscale Fourier transform infrared spectroscopy and IR imaging. Chiral nematic self-organization was observed in films from CNCs isolated at 64 wt % but not from the more heterogeneous CNC qualities produced at 58 wt %. All films degraded to some extent under simulated sunlight trials, but these effects were less pronounced in lignin-NP-containing films, suggesting a protective feature, but the hemicellulose content and CNC crystallinity may be implicated as well. Finally, heterogeneous CNC compositions obtained at a high yield and with improved resource efficiency are suggested for specific nanocellulose uses, for instance, as thickeners or reinforcing fillers, representing a step toward the development of application-tailored CNC grades.

## Introduction

Crystalline cellulose materials can be grouped into two broad categories: microcrystalline cellulose (MCC) and nanocellulose, comprising cellulose nanofibrils (CNFs), cellulose nanocrystals (CNCs), and bacterial nanocellulose.^1^ MCC and CNCs are similarly isolated from cellulose-containing feedstocks and have properties that overlap. Both materials are crystalline, chemically inert, water-dispersible, and have absorbent, strengthening, emulsifying, and viscosifying properties,^1^ with CNCs usually cited as having diameters of <10 nm and lengths of 50–350 nm,^2^ far smaller than the MCC regulatory definition of fewer than 10% of particles smaller than 5 μm.^3^

Isolation conditions tend to favor one or the other form of these cellulose crystallites; however, routes that yield colloidally stable mixed compositions of CNCs and MCC may be useful. The balance of nanomaterial and larger aggregates gives rise to the bulk properties of a given cellulose nanomaterial suspension and can be shifted to promote different property outcomes, e.g., in relation to rheology, drainage, gas barrier, swelling, optics, and mechanical properties.^4,5^ Thus, consideration of functional properties may favor the usage of dimension-spanning nanomaterials, such as mixed CNC/MCC suspensions, containing enough “nano” to enable the required properties, but perhaps providing additional benefits from micron-sized particles. Specifically, in this work, we compare how mixed CNC/MCC suspensions and films compare to standard 100% nanoscale CNC suspensions in terms of crystallinity, morphology, and colloidal stability, asking how these fundamental features translate into meaningful material properties, such as suspension rheology, self-assembly, and resilience to UV degradation, and whether inhomogeneous crystalline cellulose qualities have value in specific usage scenarios.

We demonstrate that by varying the conditions of sulfuric acid hydrolysis, it is possible to obtain a crystalline cellulose material that is broadly dimension-spanning and, consequently, property spanning within a single composition. This “bad” quality CNC suspension (if quality is gauged by material homogeneity) consists of CNCs and MCC, simultaneously produced at higher yields and under milder hydrolysis conditions than typical CNC qualities, highlighting potential environmental and economic advantages. Furthermore, we use nanoscale infrared imaging for the first time to identify lignin nanoparticles (NPs) in the compositions produced from unbleached wood pulps as well as address the UV aging of different hybrid films using a home-built solar-illumination device.

## Experimental Section

### Pulping

Norway spruce (*Picea abies*) chips, composed by mass of 44% cellulose, 17% galactoglucomannan, 6% xylan, 31% lignin, and 1% extractives, were oven dried (o.d.) and delignified in batches of 250 g in steel autoclaves (2.5 dm). The cooking liquor was added to an effective alkali charge of 22%, a sulfidity of 35%, and a liquor-to-wood ratio of 5 L/kg. The autoclaves were placed in a glycol bath at 25 °C, and the temperature was ramped at 5 °C/min to 100 °C and kept at 100 °C for 30 min to ensure impregnation. Next, the temperature was increased by 5 °C/min to 160 °C. Kraft pulping was terminated after a cooking time corresponding to an H-factor of 1600.

Part of the pulp was further delignified by oxygen in Teflon-coated steel autoclaves at a pulp consistency of 12% with 0.5% MgSO_4_ and 2.5% NaOH at 100 °C for 105 min. Bleaching was performed in plastic bags in a sequence starting with chlorine dioxide (D), followed by alkaline extraction (E), and a second D stage (Table S1), with washing with deionized water after each stage. The kraft pulp and oxygen delignified pulps are referred to in the subsequent text as unbleached softwood kraft pulp (UBSK) and bleached softwood kraft pulp (BSK), respectively.

### Cellulose Nanocrystal Isolation

Pulps were dried overnight at 50 °C and milled through a 2 mm sieve using a Thomas model 4 Wiley Mill. Hydrolyses were conducted for 30 min at 55 °C using either 58 or 64 wt % sulfuric acid. An additional 64 wt % sulfuric acid hydrolysis for 45 min at 45 °C was performed on cotton (Whatman Filter Aids, referred to as COT). All hydrolyses used a ratio of 40 g of o.d. cellulose to 17.5 mL of acid. Reactions were quenched by their addition to chilled water at 10-fold volume and then allowed to settle, with the upper clear phase discarded and the lower phase collected and rinsed by centrifugation (10 min cycles, 3600*g*). Next, the collected material was dialyzed extensively until a constant suspension pH was reached and homogenized by 1 pass through a microfluidizer at 1700 bar (M110-EH, Microfluidics). After microfluidization, the yield of the material was determined from the solid content (gravimetric concentration in wt %) and the total amount of material that was collected.

### Compositional Analysis

Prior to analysis of carbohydrate content, samples were extracted with acetone according to SCAN-CM 49:03 and then ground through a 40-mesh grid. Next, the samples were subjected to acid hydrolysis according to SCAN-CM 71:09. The monosaccharides were identified using ion chromatography coupled to a pulsed amperometric detector (IC-PAD). The acid-insoluble residue, denoted Klason lignin, was determined according to TAPPI T222 om-11 and acid-soluble lignin according to TAPPI UM 250. The total lignin content was calculated as the sum of acid-insoluble and acid-soluble lignins.

### Atomic Force Microscopy

A MultiMode 8 (NanoScope V controller) atomic force microscope (Bruker, Santa Barbara, CA) was used in PeakForce tapping mode with ScanAsyst-Air cantilevers (Bruker, Santa Barbara, CA). Using spin-coating, freshly cleaved mica surfaces were coated with polyallylamine hydrochloride (1 g/L), followed by rinsing in Milli-Q water and coating with dilute suspensions (0.01 g/L).

### Nanoscale Infrared Imaging and Spectroscopy

A scattering-type scanning near-field optical microscope (s-SNOM) from Neaspec GmbH (Germany) was used to acquire nanoscale infrared imaging and spectroscopy (nano-FTIR) spectra and infrared images. In the setup, atomic force microscopy (AFM) provided topographical images of the sample surfaces in tapping mode. The tapping amplitude was maintained at 45 nm when the tip was at the sample surface. The metal alloy coating of the AFM tip enables nano-FTIR spectroscopy and single wavenumber infrared pseudo-heterodyne imaging; a detailed description of these methods can be found in the literature.^6,7^ The pseudo-heterodyne detection technique provides a background-free accumulation of near-field optical images, with the “amplitude” image relating to reflectivity and the “phase” image relating to infrared absorption. These optical images are acquired simultaneously while obtaining the AFM topography image.

The complex scattering s-SNOM coefficient can be obtained via Fourier transform of the interferogram of the signal for each harmonic *n* of the vibration of the AFM tip, with the fourth harmonic used in this study. For the s-SNOM images, a scanning speed of 7 ms per pixel was employed, and the AFM tip was illuminated by a quantum cascade laser (QCL) at 1515 cm^–1^ (peak maximum of lignin) set to 0.25 mW output power.

The nano-FTIR spectra were obtained using a tunable femtosecond broadband laser (at repetition frequency 80 MHz, output power 0.3 mW, spectral range 1050–2000 cm^–1^), which generates the broadband IR light using difference frequency generation crystals. The spectra were collected at a resolution of 12 cm^–1^, with an average of 10 scans and a 9.8 ms integration time per scan. To compensate for the wavenumber-dependent laser energy, absorption from ambient air, optical components of the system, etc., the nano-FTIR spectra were normalized to a background spectrum obtained using a reference calibration grating (TGQ1). AFM and s-SNOM images were plane-leveled using the Gwyddion software (v. 2.55 for Windows—Czech Metrology Institute, Czech Republic).^8^

### Colloidal Properties

Surface charge was measured by conductometric titration, as has been reported elsewhere.^9^ A Zetasizer Advance (Malvern) was used to measure the DLS size and electrophoretic mobility from CNC suspensions of 0.01 and 0.1 wt %, respectively, adjusted to 10 mM NaCl. Suspensions were filtered (0.45 μm, PVDF) prior to measurement. Colloidal stability was determined by high-speed centrifugation (1000*g*; 15 min) of diluted samples (0.02 wt %). The dry content of the collected supernatant (o.d. at 105 °C) relative to the initial solid content gave the colloidal stability.

### Viscosity

Shear viscosity was measured at 25 °C using a Malvern Kinexus Pro rheometer in cone and plate geometry (a PL65 S1240 SS plate and a CP4/40 SR0734 SS cone).

### Film Making

Acid-form suspensions (0.4 wt %) were poured into Petri dishes (9 cm diameter) and evaporated at 50% RH and 23 °C. The target grammage of the films was 30 g/m^2^.

### UV–Vis Spectroscopy

The UV–vis absorbance of the films was measured using a PerkinElmer PDA UV/vis Lambda 265 spectrophotometer.

### Polarized Optical Microscopy

A Zeiss Axioplan microscope was used to image the films under crossed polarization, with a red waveplate inserted between the sample and the analyzer.

### Scanning Electron Microscopy

Film surfaces and fracture cross-sections were imaged using a Quanta 250 FEG ESEM from FEI Instruments, coupled to a X-Max 50 mm^2^ EDS from Oxford Instruments. Samples were mounted using carbon tape and imaged under high vacuum using a 2 kV beam and an Everhart–Thornley detector.

### Thermogravimetric Analysis

Thermal properties of acid-form CNC films were measured in nitrogen and air atmospheres by thermogravimetric analysis (TGA) using a TA Instruments Q5000 instrument. The temperature was first ramped at 50 °C/min to 105 °C, where it was then held isothermally for 10 min (nitrogen), followed by a 10 °C/min ramp to 1000 °C (either nitrogen or air). All samples were measured in duplicate, and reported values are an average of the two measurements.

### Wide-Angle X-ray Scattering

An Anton Paar SAXSpoint 2.0 system equipped with a Microsource X-ray source (Cu K_α_ radiation, wavelength 0.15418 nm) and a Dectris 2D CMOS Eiger R 1 M detector with a 75 μm × 75 μm pixel size was used for X-ray measurements. The beam diameter was approximately 500 μm, the sample stage temperature was 25 °C, and the beam path pressure was 1–2 mBar. Film samples were attached to a multi-solid-sample holder and mounted onto a heated sampler and a VarioStage (Anton Paar, Graz, Austria). The sample-to-detector distance was 110.8 m, and six frames of 20 min duration were read from the detector for each sample (120 min measurement time per sample). Transmittance data was recorded and used to scale the scattering intensities. SAXSdrive version 2.01.224 (Anton Paar, Graz, Austria) was used to control the instrument and SAXSanalysis version 3.00.042 (Anton Paar, Graz, Austria) for data processing. The wide-angle X-ray scattering (WAXS) diffractograms were processed (baseline correction and Gaussian fit) to obtain lattice parameters and sample crystallinities, and the Scherrer equation was used to estimate crystallite sizes.

### Attenuated Total Internal Reflectance-Fourier Transform Infrared Spectroscopy

A Spectrum One Fourier transform infrared (FTIR) spectrometer (PerkinElmer, model 73271) equipped with a universal ATR sampling accessory (PerkinElmer) and diamond crystal was used to collect attenuated total internal reflectance-Fourier transform infrared (ATR-FTIR) spectra of the CNC films (average of 16 scans, 4 cm^–1^ resolution).

### Solar Aging

CNC film stability was evaluated under 1 Sun (corresponding to AM1.5G spectrum) in the UV and visible spectral ranges. An Atlas XLS + solar simulator with static horizontal and perpendicular exposure coming from a xenon lamp (model NXE 1700) was used, and the total experiment duration was 500 h with a cumulative radiant exposure of 117 034 kJ/m^2^. The light spectrum of the lamp used in the XLS+ is reported in the literature.^10^ The ambient temperature in the chamber was approximately 36 °C, and the Black Standard Temperature sensor was 60 °C. The actual temperature of the samples ranged between these two temperatures.

Photographs were used to assess color changes (Sony A7 MK2 camera with a Laowa 100 mm f/2.8 macro 2× lens). A custom-built photograph chamber with gray interior walls and circular LED lighting (LED Neon Flex N-6x12-z-11W-40k-01, 4000K color temperature, and 1150 lm luminous flux) was used to provide even illumination. The camera was operated as follows: f/11.0 aperture, ISO 200 sensitivity, 1/20 s shutter speed, manual focus, and RAW image type. A photograph of the X-Rite ColorChecker Passport card for a white balance reference was obtained at the start of each session. Photographs were imported into Adobe Photoshop Lightroom Classic (10.3 Release), and the white balance and color profile (made using the ColorChecker passport) were applied. After a preliminary check of RGB values, exposure adjustments were applied where necessary. Subsequently, the images were exported to the JPEG format (100% quality and Adobe RGB color space). Three different non-reflecting regions were evaluated for accurate RGB measurements, with the average values of the R, B, and G pixels of these areas analyzed using MATLAB (R2021a Update 2).

## Results and Discussion

The influence of sulfuric acid concentration on the properties of CNCs hydrolyzed from unbleached (UBSK) and bleached softwood kraft (BSK) pulps was evaluated, specifically 58 wt %, previously identified as optimal in terms of yield from bleached hardwood kraft pulps,^11,12^ and 64 wt %, commonly used to produce colloidally stable CNC suspensions.^13^ In addition to the wood pulp CNCs, we produced CNCs from cotton (COT) using a classic condition from the literature in order to provide some sort of control as well as a comparison to the wood-based CNCs. The hydrolysis and purification approach followed typical steps, except instead of probe sonication, the CNCs were dispersed by microfluidization, which has been demonstrated in a few other instances.^14,15^ Diminished crystallinity with increasing pass numbers through a microfluidizer was reported for cellulose nanowhiskers isolated using a maleic acid-treatment,^16^ which may have implications for works that employ microfluidization. Hydrolysis at 58 wt % yielded non-uniform mixtures with a sediment at the bottom (see Figure S1), but once dialyzed and microfluidized, the suspensions became uniform and thick, staying this way for >1 year. This sediment is what others have referred to as “cellulose solid residue” (CSR), which is usually then separated from the CNC fraction by centrifugation,^12,14^ but can also be processed without fractionation to give cellulose nanomaterials, as was reported for maleic-acid isolated materials.^16^ Similarly, in this work, partially hydrolyzed cellulose was processed together with CNCs to give the final suspensions without any fractionation.

In Table 1, CNCs are denoted by their source (UBSK/BSK/COT) and the concentration of sulfuric acid used in the hydrolysis (58/64 wt %). After hydrolysis, the CNCs become enriched in cellulose and depleted in hemicellulose, with the lignin content of the UBSK CNCs dependent on the extent of cellulose/hemicellulose degradation. From Table S2, discrepancies between UBSK and BSK CNCs are mainly related to Klason lignin content, measured as the acid-insoluble residue. Excluding lignin, the carbohydrate compositions are quite similar for the wood CNCs hydrolyzed using the same acid concentration and reflect higher hemicellulose degradation at 64 wt % (Table S3). COT 64 has the highest cellulose content at approx. 99%, with xylose and mannose contents below 0.5% (Table S3).

**Table 1:** Relative Compositions of Starting Pulps (UBSK/BSK) and CNCs (UBSK/BSK 58/64, COT 64), Crystallite Size, and Sample Crystallinity from WAXS Analysis with Errors Included in Parentheses, and CNC Yield and Solid Content^a^

	glucose %	xylose %	lignin %	crystallite size (nm)	sample crystallinity (%)	yield %	solid content %
UBSK pulp	78.9	5.8	7.1				
UBSK 58	89.2	2.4	6.0	5.5 (0.49)	55 (1.8)	58	1.6
UBSK 64	81.6	1.0	16.6	5.2 (0.43)	46 (1.2)	11	0.3
BSK pulp	84.4	7.2	0				
BSK 58	93.3	3.0	1.1	5.6 (0.53)	52 (1.2)	64	1.4
BSK 64	96.8	1.1	1.3	5.10 (0.4)	54 (0.7)	17	0.6
COT 64	92.8	0.3	6.4	7.0 (1.06)	63 (0.8)	56	1.5

a Compositions are averages of duplicate results.

All hydrolysis conditions yielded crystalline cellulose with nearly identical lattice spacings (see Tables 1, S4, and Figure S2); however, COT 64 CNCs had the most intense scattering profile, the largest crystallite size at 7.0 nm, and the highest sample crystallinity at 63%. In general, for wood CNCs, smaller crystallites were obtained from 64 wt % and were similar in size at a given acid concentration, whether the pulp was bleached or not. UBSK 58 and BSK 58/64 had similar sample crystallinities (52–55%), whereas UBSK 64 had a lower crystallinity lower at 46%. This suggests that the lignin content of UBSK 58 does not significantly contribute to the scattering profile; however, for UBSK 64, with the higher lignin content, it becomes non-trivial to decouple non-crystalline cellulose contributions and background scattering from non-cellulose amorphous contributions.^17^

Hydrolyses at 58 wt % sulfuric acid resulted in significantly higher yields related to less overall degradation at this milder condition (Table 1). The difference in yield between UBSK 58 and UBSK 64 accounts for lignin content discrepancies since the more cellulose and hemicellulose that are degraded and dissolved, the greater the fraction of residual lignin. The yields of UBSK/BSK 58 were lower than the ∼70% reported from bleached kraft hardwood hydrolyzed using a similar approach,^12^ perhaps related to differences in source and processing. COT 64 had a relatively high yield of 56%, especially compared to <20% for UBSK/BSK 64, which may relate to the lower hydrolysis temperature and larger crystallite size (Table 1 and Figure S2). The COT 64 yield is very close to the 58% yield reported by Kloser and Gray for cotton CNCs under similar conditions.^18^ Finally, we note that while the yields of BSK 58 and BSK 64 were quite different (Table 1), this was not reflected in the sample crystallinities, which were very close at 52 and 54%, respectively.

Generally, lignin contents appear inflated, which is evident upon consideration of COT 64, which is not expected to contain appreciable lignin (Tables 1 and S2). Lignin content is determined in part from the insoluble solids remaining after acid hydrolysis and may therefore be overestimated by cellulose fragments that are resistant to acid degradation, perhaps most relevant for COT 64, with the highest relative cellulose crystallinity and crystallite size (Table 1).

From Table 1, UBSK/BSK 58 and COT 64 were all obtained at ∼1.5 wt %, whereas UBSK/BSK 64 had solid contents below 1 wt %. Not only are these samples obtained at the lowest yields but also at the most dilute concentrations. These aspects can be important from a resource usage vantage since less acid is used to produce the UBSK/BSK 58 at higher yield, requiring less water to remove residual acid and resulting in suspensions that are more concentrated. The COT 64 sample stands out again, with a solid content that is comparable to the softwood kraft pulps hydrolyzed at 58 wt %.

Table 2 presents colloidal properties, including the surface charge of different CNCs. Overall, surface charge values generally correlated to the solid content achieved after dialysis, with lower surface charge samples obtained at higher solid contents. Unbleached CNCs (UBSK 58/64) have a higher surface charge compared to the bleached CNCs (BSK 58/64), perhaps due to a charge contribution from the lignin. A surface charge of 278 mmol/kg was previously reported for CNCs from unbleached kraft softwood isolated at similar conditions to BSK 64,^19^ with the exception of the hydrolysis time and temperature, which were 45 min and 45 °C, compared to 30 min and 55 °C in the current work.

**Table 2:** Colloidal Properties of Different CNCs^a^

	surface charge (mmol/kg)	*Z*-average (nm)	electrophoretic mobility (μm·cm/V·s)	% colloidal stability	aspect ratio^b^	surface charge density (e/nm^2^)^c^
UBSK 58	179 ± 15	>400 nm*	–2.12 ± 0.06	∼100	47	0.18
UBSK 64	380 ± 15	112 (0.192)	–2.22 ± 0.08	∼100	23	0.40
BSK 58	148 ± 22	>500 nm*	–1.96 ± 0.07	∼95	40	0.15
BSK 64	279 ± 39	57.8 (0.193)	–2.65 ± 0.05	∼100	27	0.27
COT 64	218 ± 7	81.2 (0.154)	–2.65 ± 0.08	∼100	13	0.42

a Surface charge and electrophoretic mobility are presented as averages with associated standard errors, and the DLS *z*-average size is presented where possible with the associated polydispersity index indicated in parentheses.

b Estimated from average AFM dimensions.

c Estimated from the average surface charge and AFM dimensions using a density value of 1.6 g/cm^3^. *Multiple populations.

**Figure 1:**
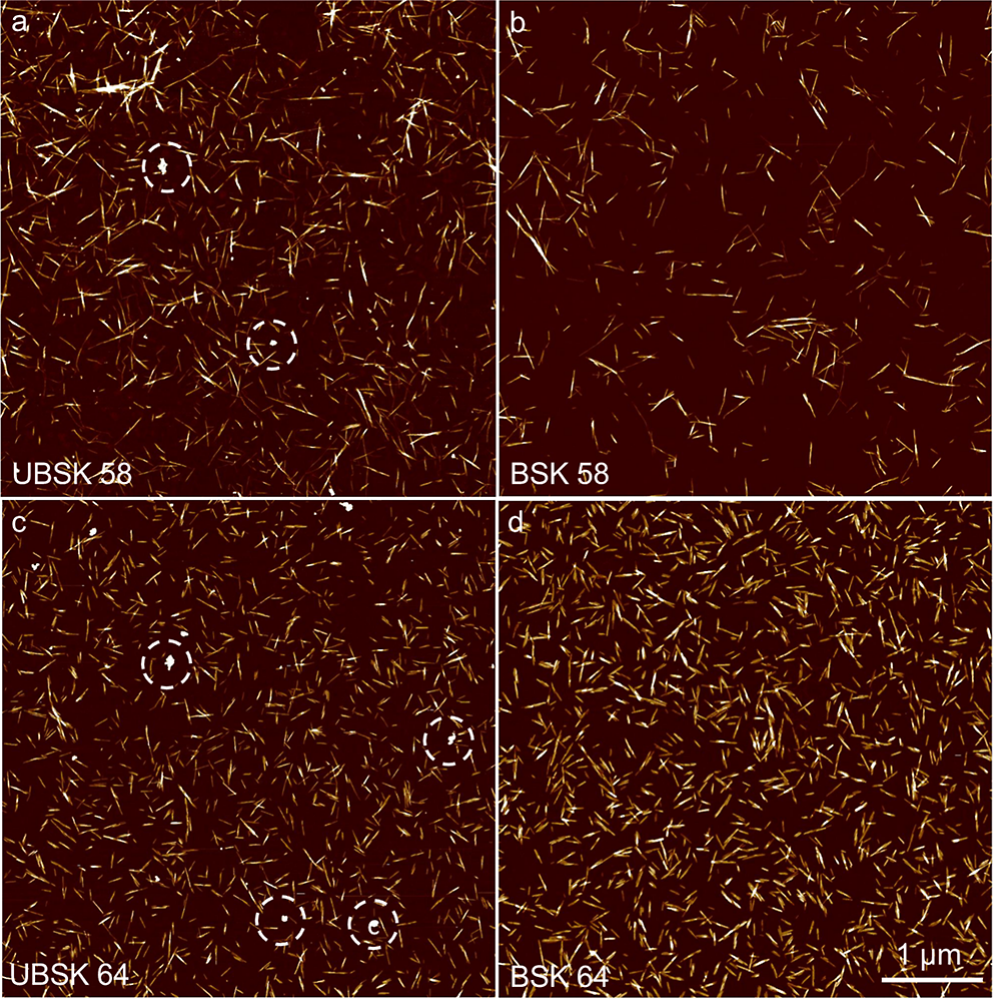
AFM height images of CNCs produced from different wood pulps (UBSK and BSK) and hydrolysis conditions (58 and 64% sulfuric acid). 10 nm *z*-scale (dark to light) and *x*–*y* scale for images (a–d) indicated in (d). Some of the L-NPs in the UBSK samples are indicated with a dashed-line circle.

Figure 1 shows AFM images of the CNCs, Figure S3 shows the length and height distributions, and Table 2 shows the aspect ratio and surface charge density calculated from average AFM dimensions. The milder hydrolyses yielded longer CNCs on average, with more polydisperse length distributions, interpreted as incomplete hydrolysis, whereas particle heights were about the same (∼4 nm) for all wood-based CNCs. The dimensions obtained at 58 wt % are similar to those previously reported for microfluidized CSR.^14^ The length distributions of the CNCs obtained from the UBSK/BSK pulps appeared alike at a given hydrolysis condition, although average lengths are longer for UBSK. Possibly, cellulose hydrolysis is less efficient in the presence of lignin since some acid is diverted toward solubilizing the lignin; however, previous work indicated that CNC dimensions are not significantly influenced by lignin.^19^ TEM sizes of bleached softwood kraft pulps hydrolyzed using 64 wt % sulfuric acid from the literature are 123 ± 58 nm × 10 ± 4 nm and 89 ± 8 nm × 5 ± 0.5 nm,^19,20^ with the former quite a bit wider than BSK 64 and the latter quite a bit shorter on average. COT 64 is similar in average length and length distribution (Figure S4) to UBSK/BSK 64; however, average COT 64 heights were ∼8 nm. Other examples of similarly isolated cotton CNCs report average AFM dimensions consistent with the current work.^9,21^

The AFM of UBSK 58/64 shows aggregates (approx. 50 nm in height), more plentiful in UBSK 64 than in UBSK 58 (Figure 1a, c). Similar aggregates are not seen in the images of the bleached CNC suspensions (Figure 1b, d), with the few small fragments that can be seen characterized by heights consistent with CNCs, below 10 nm. These aggregates are hypothesized to be L-NPs, formed in situ by the precipitation of acid-solubilized lignin during the post-hydrolysis dilution in water. This observation is consistent with a recent work that generated L-NPs in the presence of either CNFs or chitosan nanofibrils, with L-NPs bound to positively charged chitosan nanofibrils via electrostatic attraction but unbound in the case of anionic CNFs.^22^

DLS *z*-average sizes (Table 2) generally reflect the measured AFM trends, where samples hydrolyzed at the milder condition are larger and more polydisperse. UBSK 64 is nearly twice as large as BSK 64, possibly related to the presence of light absorbing/scattering L-NPs. COT 64 has a larger *z*-average size compared to BSK 64, which may be related to the larger lateral size of the cotton CNCs. Electrophoretic mobilities generally mirror surface charge values, but the UBSK CNC values may be somewhat reduced since all samples were measured in the acid-form, and the conductometric titration of these samples indicated weak acid charge groups. The centrifugation assay used to address CNC colloidal stability (Table 2) indicated excellent stability with little to no precipitation after high-speed centrifugation.

**Figure 2:**
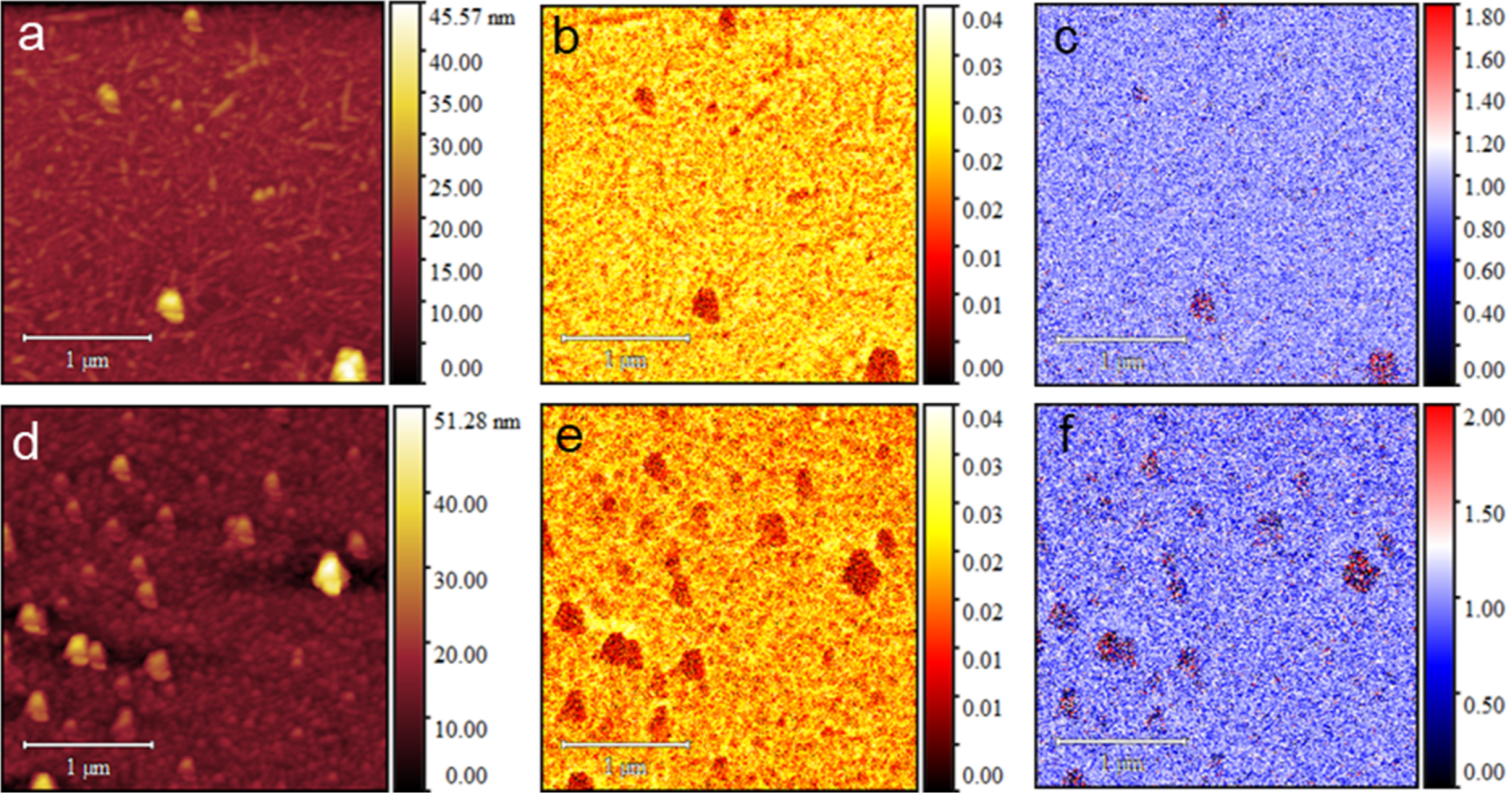
Representative AFM topography maps 3 × 3 μm (a,d), s-SNOM 4th harmonic optical amplitude image (a.u.) (b,e), and s-SNOM 4th harmonic optical amplitude phase image (rad.) (c,f) of UBSK 58 (upper row) and UBSK 64 (lower row). The s-SNOM images were acquired using the QCL laser tuned to emission at 1515 cm^–1^. Image scale bars are 1 μm.

Nano-FTIR spectroscopy and s-SNOM imaging were conducted to validate the composition of the roundish aggregates seen by AFM. First, the particles were characterized using nano-FTIR spectroscopy, with typical nano-FTIR spectra shown in the Supporting Information (and Figures S5–S7). Although the signal-to-noise ratio is fairly low due to the small size of the NPs, all spectra showed characteristic lignin IR absorbance at around 1515 cm^–1^ present in conventional lignin IR spectra and assigned to ring stretching vibrations.^23,24^ In some spectra, lignin bands at around 1430 and 1460 cm^–1^ are also seen.^25^ Nano-IR spectra of L-NPs in the characteristic cellulose absorption region at about 1100 cm^–1^ show no absorption (Figure S8), indicating that the NPs primarily consist of lignin with a cellulose content below detection. Additionally, conventional ATR-FTIR (Figure S9) of UBSK CNC films showed only cellulose vibrations (attributed to relatively low lignin contents and the bulk nature of the measurement); thus, nanoscale IR investigations were needed to confirm lignin. Finally, taking the AFM (Figure S3) and nano-FTIR results together, we posit that the lignin in these materials is mainly in the form of independent lignin NP colloids and not as a coating on CNC surfaces, as is sometimes referred to as L-CNCs.

s-SNOM was used to study the distribution of lignin in UBSK 58/64 using the lignin absorption at 1515 cm^–1^. The acquired s-SNOM optical images reveal heterogeneity both in the amplitude (Figure 2b,d) and phase (Figure 2e,f). Compared to the highly reflective surface of the Si wafer, all particles (higher features in the AFM topography images in Figure 2a, c) exhibit lower reflectivity in the amplitude images. In contrast, only the L-NPs show a higher phase corresponding to an enhanced IR absorption at 1515 cm^–1^. Moreover, the AFM and s-SNOM images in Figure 2 correspond very well to each other, confirming that the particles are lignin-based.

**Figure 3:**
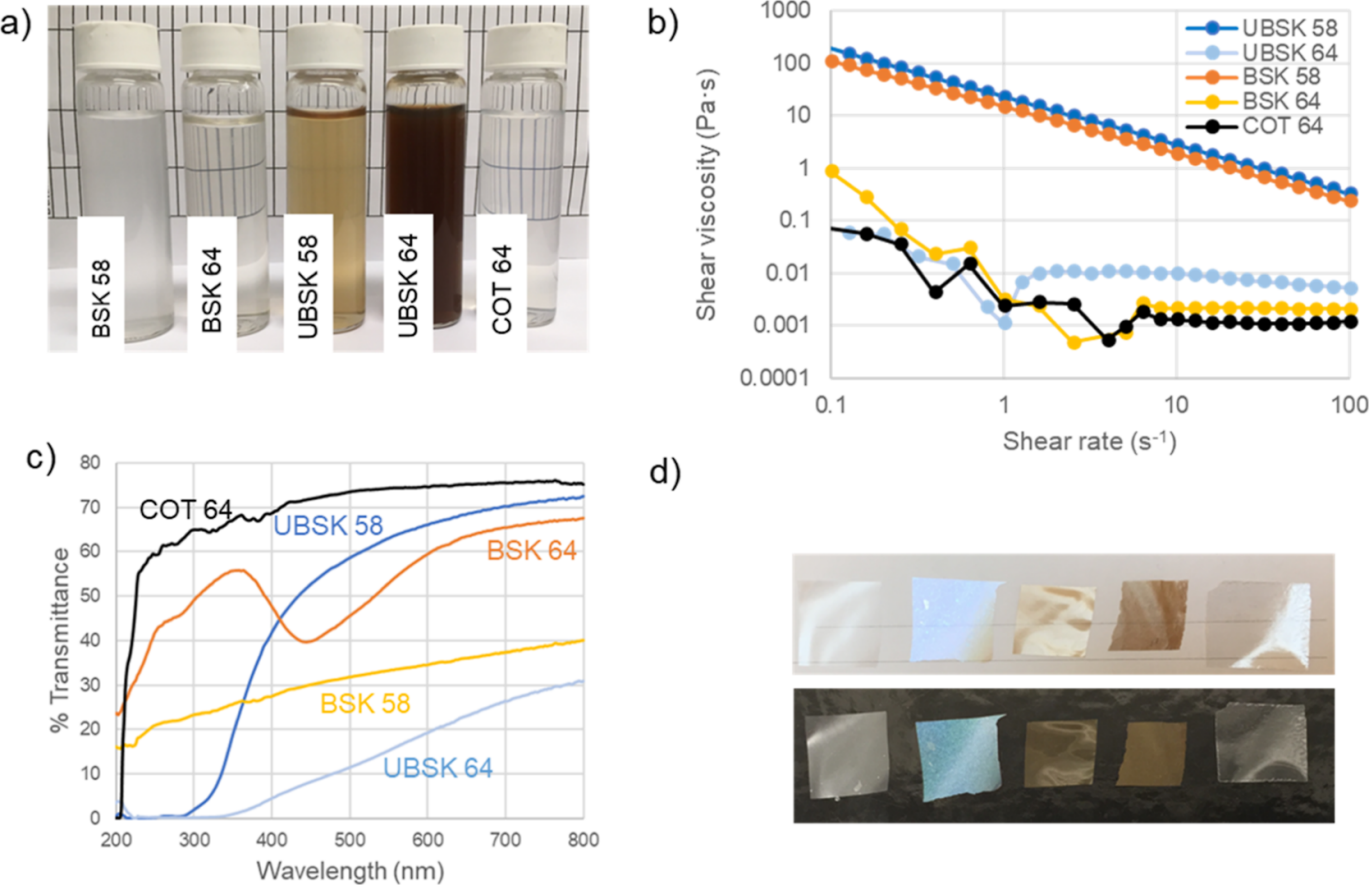
Photographs of CNC suspensions at 0.5 wt % (a) shear viscosity of suspensions at 1.75 wt %; (b) transmittance spectra of films; (c) photographs of film pieces (∼2 cm^2^, 20 μm-thick) on white and black backgrounds (d). The suspensions and films (a,d) are arranged in the same sequence from left to right: BSK 58, BSK 64, UBSK 58, UBSK 64, and COT 64.

UBSK suspensions appear brown in color due to the presence of lignin, with UBSK 64 being darker due to its higher lignin content (Figure 3a). UBSK/BSK 58 suspensions appear somewhat hazy (Figure 3a), consistent with the large DLS *z*-average sizes, and significantly more viscous compared to UBSK/BSK/COT 64 (Figure 3b). The higher viscosity and more pronounced shear thinning of UBSK/BSK 58 are consistent with their lower charge and higher aspect ratio.^19,26,27^ Conversely, UBSK/BSK/COT 64 shows unstable profiles at low shear and little shear thinning. L-NPs seem to slightly increase shear viscosity, although the reason is not obvious since the lignin-containing CNCs contain less nanocellulose at a given total solid content. The viscosity profiles of UBSK/BSK 58 CNCs may be beneficial for certain applications, for instance, as thickeners, where achieving a higher viscosity at a lower additive content is attractive.

Including lignin in nanocellulose is reported to improve hydrophobicity and to impart antioxidant and UV-absorbing properties.^20,22,28−33^ Films cast from different suspensions are shown in Figure 3d, and the UV–vis spectra of these films are shown in Figure 3c, with COT 64 being the most transparent, BSK 64 having a chiral nematic reflection at ∼445 nm, UBSK 64 absorbing UVA, UVB, and a significant amount of UVC (<5% transmittance) but also being the most opaque due to its dark color, and UBSK 58 having intermediate properties, with very low transmittance of UVA and UVB increasing into the UVC and reasonably high transmittance across the visible spectrum (∼40–70%; wavelength dependent). Interestingly, the BSK 58 film is significantly less transparent than UBSK 58 (excluding the lignin absorbing spectral region) and BSK 64 films, the former possibly due to L-NPs filling voids and the latter to increased scattering. Films produced from chitin nanofibers with different L-NP contents (9–23%) showed similar optical profiles to the lignin-containing films in this work.^22^

**Figure 4:**
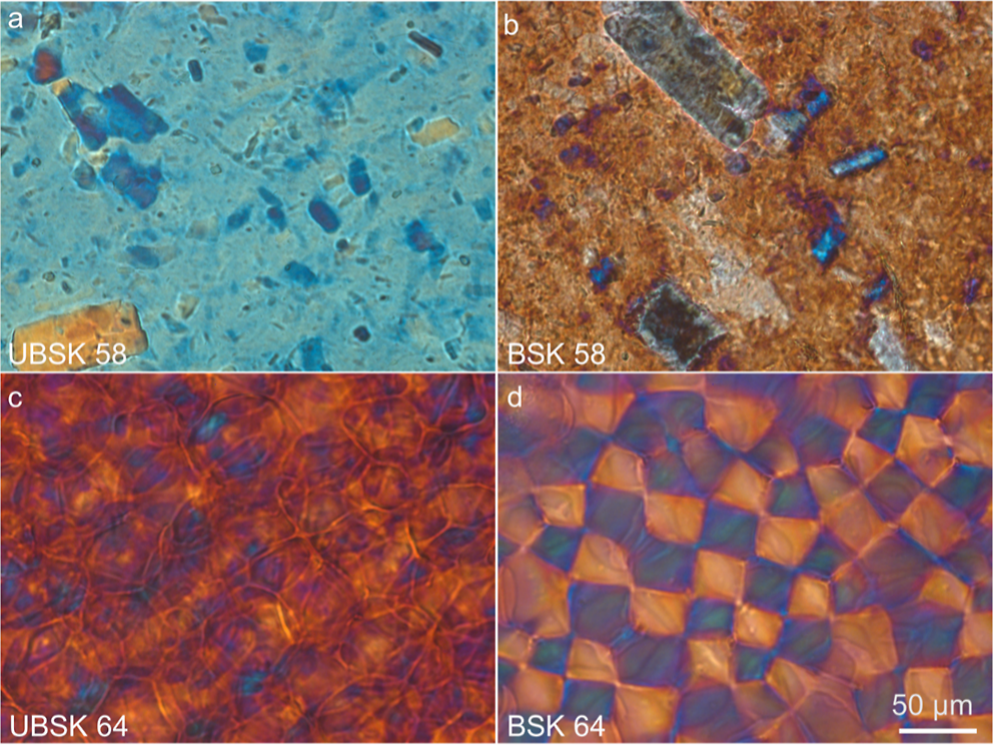
Polarized optical microscopy images of CNC films prepared from different pulp types (UBSK and BSK) and sulfuric acid concentrations (58 and 64 wt %). Figures (a,c) are films cast from UBSK 58 and UBSK 64 CNCs, respectively, and (b,d) are the BSK counterparts. The scale bar for images (a–d) is indicated in (d).

**Figure 5:**
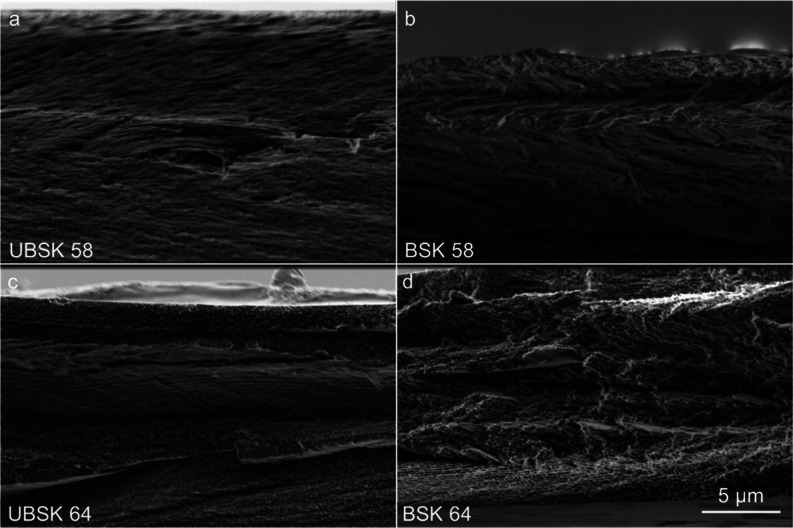
Cross-sectional SEM images of CNC films; figures (a,c) are of UBSK 58 and UBSK 64 films, respectively, and (b,d) are the BSK counterparts. The scale bars for images (a–d) are indicated in (d).

By POM, UBSK/BSK 58 films present large (10–100 s of μm) birefringent particles on a largely monochromatic birefringent backdrop (Figure 4a,b). These large crystallites are also visible by scanning electron microscopy (SEM) of film surfaces (Figure S10a,b) but not obviously within filmcross-sections (Figure 5a,b), which appear compact and stratified, suggesting that the large crystallites are securely embedded within the bulk. The birefringence and size of these particles (Figure 4a,b) supports that they are partially hydrolyzed cellulose fragments in the form of MCC, and the monochromatic background observed in cross-sectional POM suggests a planar alignment of the surrounding CNCs (Figure S11b). We see no evidence of chiral nematic assembly in these films, probably due to polydispersity and large crystallites (MCC) interfering with CNC self-organization. MCC is responsible for the haziness captured in Figure 3a and the higher yield achieved using the lower acid concentration. The amount of MCC relative to CNC was not measured, but the CNC fraction seems to dominate in the POM (Figure 4). To verify the claim of MCC, we observed a commercial MCC sample in dilute dispersion, which showed birefringent particles similar in appearance and size to the μm-sized particles in the UBSK/BSK 58 films (Figure S11a). What is not obvious is whether these large MCC-like aggregates are present in the suspension from the start or form as the suspensions are concentrated into films (no obvious aggregates were seen in suspension POM).

POM of the UBSK 64 film (Figure 4c) shows a birefringent rosette-like structure with the rosettes separated by densified walls, also seen by SEM (Figure S10c) and a chiral nematic fingerprint motif apparent in higher magnification POM (Figure S12a) and in cross-sectional SEM (Figure 5c). However, UBSK 64 had no chiral nematic reflection by UV–vis (Figure 3c), perhaps because its pitch is obscured by lignin absorbance. Indeed, others have observed protein adsorption bands that overlapped with the short-wavelength reflection bands of chiral nematic structures.^34^ BSK 64 has a parabolic focal conic liquid crystalline texture (Figure 4d), a structure that was first reported in CNC films by Roman and Gray.^35^ Additionally, BSK 64 POM (Figure S12b) and cross-sectional SEM (Figure 5d) show chiral nematic organization that is also confirmed by visible iridescence (Figure 3d). COT 64 film cross-sections by SEM (Figure S12d) show regular chiral nematic organization, with the chiral nematic director nearly perpendicular to the film surface, whereas by POM, this film appears comparatively featureless, consistent with the in-plane CNC alignment (Figure S12c). This chiral nematic film does not have a reflectance band in the visible range since its pitch is red shifted outside the visible range (Figure S12d). Overall, it seems that hydrolysis using 64 wt % sulfuric acid gives CNCs the right properties for chiral nematic self-assembly, whereas it seems that hydrolysis at 58 wt % does not support chiral nematic self-assembly.

Thermal profiles were similar for all CNCs (Figure S13), both in air and in nitrogen (not shown), with thermal properties in the air and nitrogen summarized in Table 3. In the air, the first thermal event at 168–175 °C is consistent with the onset of degradation of acid-form CNCs, eventually giving a charred residue, which was further degraded and oxidized with increasing temperature, whereas in nitrogen, the char was carbonized, such that approximately ∼30% carbon remains at 1000 °C (slightly higher from L-NP-containing CNCs). Overall, the presence of MCC or L-NPs or variations in hydrolysis conditions did not significantly influence the degradation onset. This contrasts with a study of bacterial CNCs that related a higher charge from sulfate half-esters to a lower degradation temperature.^36^ Another study reported that counterion exchange to sodium-form eliminates the dependency of thermal stability on surface charge for sulfuric acid-hydrolyzed CNCs.^37^ However, the CNCs studied in the current work are all in acid-form and present a range of surface charges (∼150–380 mmol/kg) but begin to degrade at very similar temperatures, implicating other parameters in the current results, such as cellulose crystallinity or aspect ratio. Furthermore, the effect of lignin on the thermal properties of cellulose materials is conflicting, with some reports relating an improvement in thermal stability,^20,32,33^ and others a decrease in the degradation onset.^3^ Discrepancies may be related to differences in lignin content, form, and chemistry, as well as the properties of the cellulose.

**Table 3:** TGA Analysis of CNC Films (Acid-Form) Heated in Air and Nitrogen, with Degradation Temperatures (*T*_deg_) of Main Thermal Decompositions and Mass % Remaining at 1000 °C Presented

	atm	dry content (%)	*T*_deg_ 1 (°C)	*T*_deg_ 2 (°C)	*T*_deg_ 3 (°C)	residue at 1000 °C (%)
UBSK 58	N_2_	97.6	173	381		29.1
	air	97.6	174	365	537	0.5
UBSK 64	N_2_	97.2	170	388		32.6
	air	97.6	173		491	1.1
BSK 58	N_2_	97.2	173	372		28.4
	air	97.4	174	357	509	0.8
BSK 64	N_2_	97.3	168	382		28.6
	air	97.3	168	370	492	1.3
COT 64	N_2_	99.1	174	391		28.2
	air	99.0	175	384	542	0.3

Lastly, film stability under conditions of continuous simulated solar exposure was evaluated to address the feasibility of using these films in scenarios where they are exposed to sunlight, e.g., photovoltaics, sensors, or coatings.^38^ We previously studied nanocellulose films integrated with inorganic UV-absorbing NPs;^39,40^ however, how the photostability of L-NPs compares is unclear. To assess aging, changes in color and hue were quantified via deconvolution of the RBG channels from digital photographs taken at different times. Figure S14 presents RGB values as a function of time for the wood-based CNC films, with all films becoming darker as indicated by a decrease in RGB values, with BSK 64 and UBSK 58/64 films retaining their color hue, whereas the BSK 58 film underwent a hue change from neutral gray to yellow at approximately 150 h. Furthermore, the BSK 58 film became lighter between the third and fourth data points, likely due to the hue change, followed by steady darkening. BSK 64 and UBSK 58/64 films underwent a relatively fast degradation in RGB values during the first 150 h, followed by a much slower color change, whereas the BSK 58 film darkened at a comparatively fast rate overall. The distinct behavior in the BSK 58 film may be related to the appearance of inhomogeneities after approximately 100 h and their growth with increasing exposure (Figures S15 and S16). We remain uncertain about the nature of these aggregates and why they form, observing only that the measured sample crystallinities of BSK 58 and BSK 64 are quite similar but that the ratio of hemicellulose to cellulose is threefold larger for BSK 58 than for BSK 64 (Table 1), perhaps implicating hemicellulose content in the distinct UV aging behavior of BSK 58. Furthermore, we also observe that these aggregates do not occur in UBSK 58 films, perhaps due to the presence of lignin in this sample. Table 4 summarizes the main results of the simulated solar aging trials, with all films presenting a visible color change, which was more pronounced in films hydrolyzed from 58 wt % sulfuric acid.

**Table 4:**
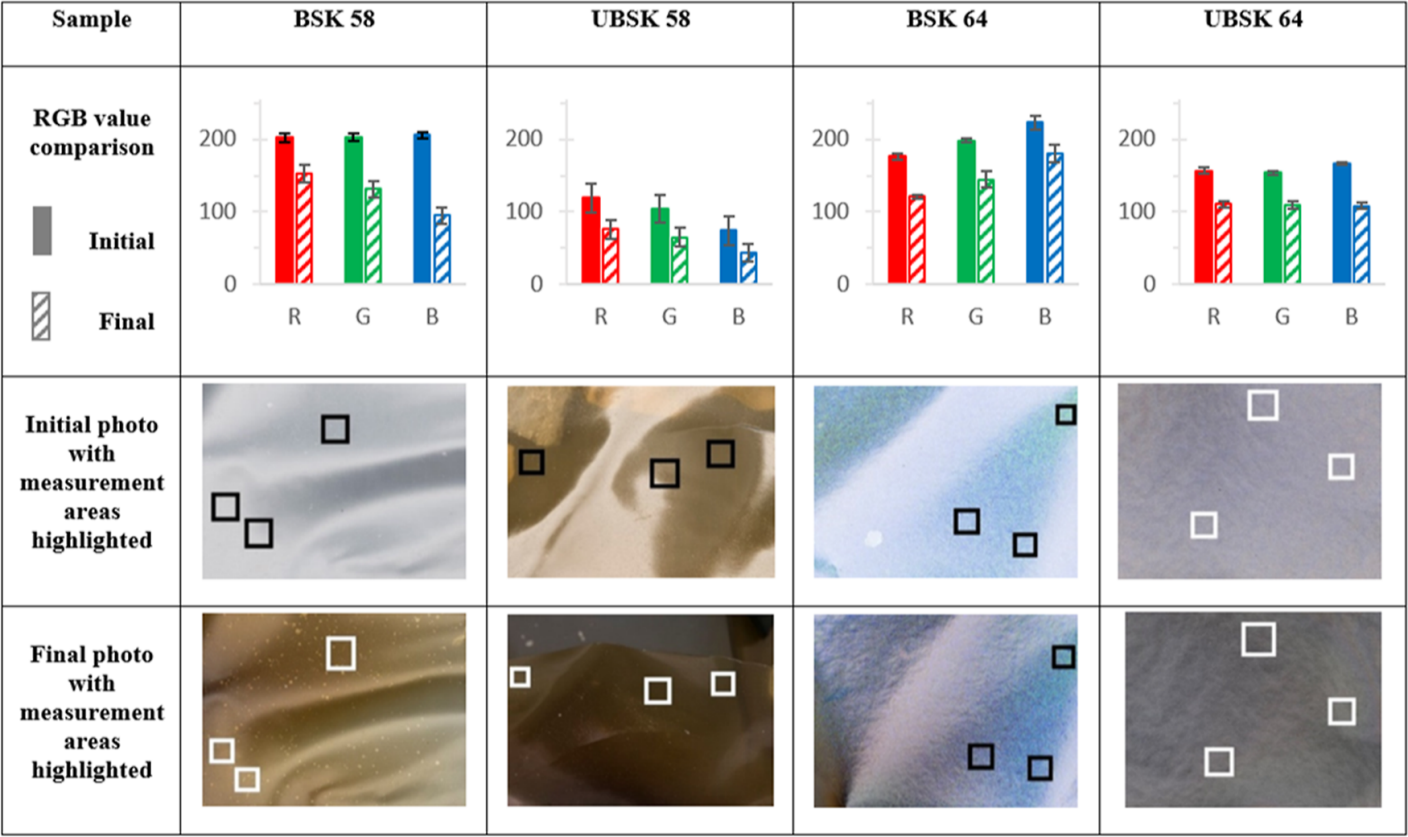
Comparison of Initial and Final RGB Values and Corresponding Photographs of CNC Films, before and after UV Exposure at a Cumulative Radiant Exposure of 117 034 kJ/m^2^ over 500 h^a^

a Error bars indicate the standard deviation between the three measurement areas.

The temperatures during this experiment fell far below the degradation temperatures measured by TGA (Table 3); however, all films underwent visible deterioration indicative of UV-driven degradation and/or thermally driven degradation/dehydration. This result diverges from similar exposure trials of structurally colored CNC films, where pure CNC films exhibited color stability.^41^ The discrepancy may be related to differences in CNC crystallinity, counterion, or exposure conditions. Indeed, compared to this work (xenon lamp, 500 h), the previous study used a halogen lamp and 1000 h, translating to 2.5× more UV exposure in the current study because the halogen lamp produces 5× less UV irradiation.

UV–vis (Figure S17) and ATR-FTIR (Figure S18) were measured before and after exposure. The UV–vis corroborated the previous analysis, specifically that films cast from CNCs hydrolyzed at 58% are more changed upon exposure, with the BSK 58 film experiencing the most pronounced darkening overall, likely related to dynamic aggregate formation. In UBSK 64, transmittance below 300 nm is unaffected by exposure as it was negligible from the start; however, after exposure, the opacity extends into the visible range (up to approx. 500 nm). The BSK 64 film initially showed a chiral nematic reflection, but this feature was obscured after exposure due to an overall increase in film opacity. The extent of the degradation may be muted by the relatively high crystallinity of cellulose in samples isolated at 64 wt % sulfuric acid. From ATR-FTIR (Figure S18), all films show intensity losses in the fingerprint region after exposure and the emergence of a new band at approximately 1720 cm^–1^. However, comparison of films produced at the same conditions of hydrolysis seems to indicate a protective value to the lignin, with less loss of IR intensity for the lignin-containing samples. Whereas a comparison of UBSK/BSK 58 to UBSK/BSK 64 shows less intensity losses overall in the samples produced from the harsher hydrolysis condition, perhaps again implicating cellulose crystallinity in the resilience to UV-degradation. Together, this data points to cellulose degradation and the formation of oxidized degradation products, but with less pronounced changes where L-NPs are present and when the cellulose is more crystalline. It seems then that lignin NPs do mitigate some of the effects of UV-exposure in CNC-based films; however, this is accomplished with a significant loss in transparency and may need even higher lignin contents to afford meaningful protection in real life settings.

## Conclusions

Cellulosic particles were isolated from bleached and unbleached softwood kraft pulps. The harsher hydrolyses resulted in CNCs, whereas the milder hydrolyses gave a heterogeneous composition consisting of CNCs and MCC. Unbleached pulp hydrolyses additionally yielded lignin NPs. Milder hydrolyses produced longer and more polydisperse CNCs at higher yield to give hazy suspensions that were significantly more viscous and shear thinning. Chiral nematic self-assembly was not observed in the CNCs produced using these conditions, likely because of their polydispersity. Whereas the exact lignin content was difficult to quantify accurately due to cellulose crystallite recalcitrance, nano-FTIR spectroscopy and IR imaging enabled conclusive compositional identification. The different CNCs had a similar onset for thermal degradation; however, L-NP-containing films better resisted UV degradation, possibly additionally related to differences in the compositional makeup, specifically hemicellulose content, and to cellulose crystallinity. Overall, CNC suspensions of varying qualities, e.g., lignin-enriched dimension spanning, may prove useful in specific implementations of nanocellulose that require CNC-like properties coupled with higher yield, less “nano”, and potential lignin-derived features, such as opacity or antimicrobial activity.

## Supporting Information Available

Bleaching protocol; images of the UBSK CNC suspensions during dialysis; compositional analysis of CNCs; WAXS diffractograms and cellulose Iβ lattice spacings; size analysis of CNCs from AFM; AFM maps; nano-FTIR spectra; ATR-FTIR spectra; POM of various samples; cross-sectional SEM of COT 64; TGA; RGB values as a function of time upon simulated solar exposure; photographs, UV–vis spectra, and ATR-FTIR spectra before and after exposure (PDF)

## Abbreviations

CNC: cellulose nanocrystal
MCC: microcrystalline cellulose
CNF: cellulose nanofibril
L-NP: lignin nanoparticle

## References

[ref1] Ventura-Cruz S.; Tecante A. Nanocellulose and Microcrystalline Cellulose from Agricultural Waste: Review on Isolation and Application as Reinforcement in Polymeric Matrices. Food Hydrocolloids 2021, 118, 106771 10.1016/J.FOODHYD.2021.106771.

[ref2] Foster E. J.; Moon R. J.; Agarwal U. P.; Bortner M. J.; Bras J.; Camarero-Espinosa S.; Chan K. J.; Clift M. J. D. D.; Cranston E. D.; Eichhorn S. J.; Fox D. M.; Hamad W. Y.; Heux L.; Jean B.; Korey M.; Nieh W.; Ong K. J.; Reid M. S.; Renneckar S.; Roberts R.; Shatkin J. A.; Simonsen J.; Stinson-Bagby K.; Wanasekara N.; Youngblood J. Current Characterization Methods for Cellulose Nanomaterials; 2018; Vol. 47, pp 2609–2679. http://xlink.rsc.org/?DOI=C6CS00895J (accessed September 25, 2018). 10.1039/c6cs00895j 29658545

[ref3] Vanhatalo K.; Maximova N.; Perander A. M.; Johansson L. S.; Haimi E.; Dahl O. Comparison of Conventional and Lignin-Rich Microcrystalline Cellulose. BioResources 2016, 11, 4037–4054. 10.15376/biores.11.2.4037-4054.

[ref4] Aulin C.; Gällstedt M.; Lindström T. Oxygen and Oil Barrier Properties of Microfibrillated Cellulose Films and Coatings. Cellulose 2010, 17, 559–574. 10.1007/S10570-009-9393-Y.

[ref5] Minelli M.; Baschetti M. G.; Doghieri F.; Ankerfors M.; Lindström T.; Siró I.; Plackett D. Investigation of Mass Transport Properties of Microfibrillated Cellulose (MFC) Films. J. Membr. Sci. 2010, 358, 67–75. 10.1016/J.MEMSCI.2010.04.030.

[ref6] Huth F.; Govyadinov A.; Amarie S.; Nuansing W.; Keilmann F.; Hillenbrand R. Nano-FTIR Absorption Spectroscopy of Molecular Fingerprints at 20 Nm Spatial Resolution. Nano Lett. 2012, 12, 3973–3978. 10.1021/nl301159v. 22703339

[ref7] Govyadinov A. A.; Amenabar I.; Huth F.; Carney P. S.; Hillenbrand R. Quantitative Measurement of Local Infrared Absorption and Dielectric Function with Tip-Enhanced Near-Field Microscopy. J. Phys. Chem. Lett. 2013, 4, 1526–1531. 10.1021/jz400453r. 26282309

[ref8] Nečas D.; Klapetek P. Gwyddion: An Open-Source Software for SPM Data Analysis. Open Phys. 2012, 10, 181 10.2478/s11534-011-0096-2.

[ref9] Abitbol T.; Kloser E.; Gray D. G. Estimation of the Surface Sulfur Content of Cellulose Nanocrystals Prepared by Sulfuric Acid Hydrolysis. Cellulose 2013, 20, 785–794. 10.1007/S10570-013-9871-0.

[ref10] Poskela A.; Miettunen K.; Tiihonen A.; Lund P. D. Extreme Sensitivity of Dye Solar Cells to UV-Induced Degradation. Energy Sci. Eng. 2021, 9, 19–26. 10.1002/ESE3.810.

[ref11] Wang Q.; Zhao X.; Zhu J. Y. Kinetics of Strong Acid Hydrolysis of a Bleached Kraft Pulp for Producing Cellulose Nanocrystals (CNCs). Ind. Eng. Chem. Res. 2014, 53, 11007–11014. 10.1021/IE501672M.

[ref12] Chen L.; Wang Q.; Hirth K.; Baez C.; Agarwal U. P.; Zhu J. Y. Tailoring the Yield and Characteristics of Wood Cellulose Nanocrystals (CNC) Using Concentrated Acid Hydrolysis. Cellulose 2015, 22, 1753–1762. 10.1007/S10570-015-0615-1.

[ref13] Dong X. M.; Revol J.-F. F.; Gray D. G. Effect of Microcrystallite Preparation Conditions on the Formation of Colloid Crystals of Cellulose. Cellulose 1998, 5, 19–32. 10.1023/A:1009260511939.

[ref14] Wang Q. Q.; Zhu J. Y.; Reiner R. S.; Verrill S. P.; Baxa U.; McNeil S. E. Approaching Zero Cellulose Loss in Cellulose Nanocrystal (CNC) Production: Recovery and Characterization of Cellulosic Solid Residues (CSR) and CNC. Cellulose 2012, 19, 2033–2047. 10.1007/S10570-012-9765-6.

[ref15] Ojala J.; Sirviö J. A.; Liimatainen H. Preparation of Cellulose Nanocrystals from Lignin-Rich Reject Material for Oil Emulsification in an Aqueous Environment. Cellulose 2017, 25, 293–304. 10.1007/S10570-017-1568-3.

[ref16] Wang H.; Zhu J. J.; Ma Q.; Agarwal U. P.; Gleisner R.; Reiner R.; Baez C.; Zhu J. Y. Pilot-Scale Production of Cellulosic Nanowhiskers With Similar Morphology to Cellulose Nanocrystals. Front. Bioeng. Biotechnol. 2020, 8, 1070 10.3389/fbioe.2020.565084. PMC7500145 33015018

[ref17] Agarwal U. P.; Reiner R. R.; Ralph S. A. Estimation of Cellulose Crystallinity of Lignocelluloses Using Near-IR FT-Raman Spectroscopy and Comparison of the Raman and Segal-WAXS Methods. J. Agric. Food Chem. 2012, 61, 103–113. 10.1021/JF304465K. 23241140

[ref18] Kloser E.; Gray D. G. Surface Grafting of Cellulose Nanocrystals with Poly(Ethylene Oxide) in Aqueous Media. Langmuir 2010, 26, 13450–13456. 10.1021/LA101795S. 20695591

[ref19] Abitbol T.; Kam D.; Levi-Kalisman Y.; Gray D. G.; Shoseyov O. Surface Charge Influence on the Phase Separation and Viscosity of Cellulose Nanocrystals. Langmuir 2018, 34, 3925–3933. 10.1021/acs.langmuir.7b04127. 29513998

[ref20] Agarwal U. P.; Ralph S. A.; Reiner R. S.; Hunt C. G.; Baez C.; Ibach R.; Hirth K. C. Production of High Lignin-Containing and Lignin-Free Cellulose Nanocrystals from Wood. Cellulose 2018, 25, 5791–5805. 10.1007/S10570-018-1984-Z.

[ref21] Kan K. H. M.; Li J.; Wijesekera K.; Cranston E. D. Polymer-Grafted Cellulose Nanocrystals as PH-Responsive Reversible Flocculants. Biomacromolecules 2013, 14, 3130–3139. 10.1021/BM400752K. 23865631

[ref22] Pasquier E.; Mattos B. D.; Belgacem N.; Bras J.; Rojas O. J. Lignin Nanoparticle Nucleation and Growth on Cellulose and Chitin Nanofibers. Biomacromolecules 2021, 22, 880–889. 10.1021/ACS.BIOMAC.0C01596. 33377786

[ref23] Barnette A. L.; Lee C.; Bradley L. C.; Schreiner E. P.; Park Y. B.; Shin H.; Cosgrove D. J.; Park S.; Kim S. H. Quantification of Crystalline Cellulose in Lignocellulosic Biomass Using Sum Frequency Generation (SFG) Vibration Spectroscopy and Comparison with Other Analytical Methods. Carbohydr. Polym. 2012, 89, 802–809. 10.1016/j.carbpol.2012.04.014. 24750865

[ref24] Sammons R. J.; Harper D. P.; Labbé N.; Bozell J. J.; Elder T.; Rials T. G. Characterization of Organosolv Lignins Using Thermal and FT-IR Spectroscopic Analysis. BioResources 2013, 8, 2752–2767. 10.15376/biores.8.2.2752-2767.

[ref25] Raspolli Galletti A. M.; D’Alessio A.; Licursi D.; Antonetti C.; Valentini G.; Galia A.; Nassi O Di Nasso N. Midinfrared FT-IR as a Tool for Monitoring Herbaceous Biomass Composition and Its Conversion to Furfural. J. Spectrosc. 2015, 2015, 1–12. 10.1155/2015/719042.

[ref26] Shafeiei-Sabet S.; Hamad W. Y.; Hatzikiriakos S. G. Influence of Degree of Sulfation on the Rheology of Cellulose Nanocrystal Suspensions. Rheol. Acta 2013, 52, 741–751. 10.1007/s00397-013-0722-6.

[ref27] Wu Q.; Meng Y.; Wang S.; Li Y.; Fu S.; Ma L.; Harper D. Rheological Behavior of Cellulose Nanocrystal Suspension: Influence of Concentration and Aspect Ratio. J. Appl. Polym. Sci. 2014, 131 40525 10.1002/app.40525.

[ref28] Teh K. C.; Foo M. L.; Ooi C. W.; Leng Chew I. M. Sustainable and Cost-Effective Approach for the Synthesis of Lignin-Containing Cellulose Nanocrystals from Oil Palm Empty Fruit Bunch. Chemosphere 2021, 267, 129277 10.1016/J.CHEMOSPHERE.2020.129277. 33385850

[ref29] Jiang J.; Carrillo-Enríquez N. C.; Oguzlu H.; Han X.; Bi R.; Song M.; Saddler J. N.; Sun R.-C.; Jiang F. High Production Yield and More Thermally Stable Lignin-Containing Cellulose Nanocrystals Isolated Using a Ternary Acidic Deep Eutectic Solvent. ACS Sustainable Chem. Eng. 2020, 8, 7182–7191. 10.1021/ACSSUSCHEMENG.0C01724.

[ref30] Gupta A.; Simmons W.; Schueneman G. T.; Mintz E. A. Lignin-Coated Cellulose Nanocrystals as Promising Nucleating Agent for Poly(Lactic Acid). J. Therm. Anal. Calorim. 2016, 126, 1243–1251. 10.1007/S10973-016-5657-6.

[ref31] Wei L.; Agarwal U. P.; Matuana L.; Sabo R. C.; Stark N. M. Performance of High Lignin Content Cellulose Nanocrystals in Poly(Lactic Acid). Polymer 2018, 135, 305–313. 10.1016/J.POLYMER.2017.12.039.

[ref32] Bian H.; Chen L.; Dai H.; Zhu J. Y. Integrated Production of Lignin Containing Cellulose Nanocrystals (LCNC) and Nanofibrils (LCNF) Using an Easily Recyclable Di-Carboxylic Acid. Carbohydr. Polym. 2017, 167, 167–176. 10.1016/J.CARBPOL.2017.03.050. 28433151

[ref33] Wang Y.; Liu S.; Wang Q.; Fu X.; Fatehi P. Performance of Polyvinyl Alcohol Hydrogel Reinforced with Lignin-Containing Cellulose Nanocrystals. Cellulose 2020, 27, 8725–8743. 10.1007/S10570-020-03396-Z.

[ref34] Bast L. K.; Klockars K. W.; Greca L. G.; Rojas O. J.; Tardy B. L.; Bruns N. Infiltration of Proteins in Cholesteric Cellulose Structures. Biomacromolecules 2021, 22, 2067 10.1021/acs.biomac.1c00183. 33899466 PMC8154265

[ref35] Roman M.; Gray D. G. Parabolic Focal Conics in Self-Assembled Solid Films of Cellulose Nanocrystals. Langmuir 2005, 21, 5555–5561. 10.1021/LA046797F. 15924489

[ref36] Roman M.; Winter W. T. Effect of Sulfate Groups from Sulfuric Acid Hydrolysis on the Thermal Degradation Behavior of Bacterial Cellulose. Biomacromolecules 2004, 5, 1671–1677. 10.1021/bm034519+. 15360274

[ref37] Vanderfleet O. M.; Reid M. S.; Bras J.; Heux L.; Godoy-Vargas J.; Panga M. K. R.; Cranston E. D. Insight into Thermal Stability of Cellulose Nanocrystals from New Hydrolysis Methods with Acid Blends. Cellulose 2019, 26, 507–528. 10.1007/s10570-018-2175-7.

[ref38] Kaschuk J. J.; Al Haj Y.; Rojas O. J.; Miettunen K.; Abitbol T.; Vapaavuori J.; Kaschuk J. J.; Rojas O. J.; Al Haj Y.; Vapaavuori J. Plant-Based Structures as an Opportunity to Engineer Optical Functions in Next-Generation Light Management. Adv. Mater. 2021, 34, 2104473 10.1002/ADMA.202104473. 34699648

[ref39] Abitbol T.; Marway H. S.; Kedzior S. A.; Yang X.; Franey A.; Gray D. G.; Cranston E. D. Hybrid Fluorescent Nanoparticles from Quantum Dots Coupled to Cellulose Nanocrystals. Cellulose 2017, 24, 1287–1293. 10.1007/s10570-016-1188-3.

[ref40] Abitbol T.; Ahniyaz A.; Álvarez-Asencio R.; Fall A.; Swerin A. Nanocellulose-Based Hybrid Materials for UV Blocking and Mechanically Robust Barriers. ACS Appl. Bio Mater. 2020, 3, 2245–2254. 10.1021/ACSABM.0C00058. 35025276

[ref41] Klockars K. W.; Yau N. E.; Tardy B. L.; Majoinen J.; Kämäräinen T.; Miettunen K.; Boutonnet E.; Borghei M.; Beidler J.; Rojas O. J. Asymmetrical Coffee Rings from Cellulose Nanocrystals and Prospects in Art and Design. Cellulose 2019, 26, 491–506. 10.1007/s10570-018-2167-7.

