# Supplementary material for: Isolation of Mixed Compositions of Cellulose Nanocrystals, Microcrystalline Cellulose, and Lignin Nanoparticles from Wood Pulps
Source: ACS Omega. 2023 May 19;8(24):21474–84. doi: 10.1021/acsomega.3c00295 (PMC10286283; doi:10.1021/acsomega.3c00295)
Supplement: Supplementary file 1 — ao3c00295_si_001.pdf [file ao3c00295_si_001.pdf]

Supporting information

Isolation of mixed compositions of cellulose nanocrystals, microcrystalline cellulose, and lignin nanoparticles from wood pulps

Tiffany Abitbol[1,2], Mikaela Kubat[2], Elisabet Brännvall[2], Nikolay Kotov[3], C. Magnus Johnson[3], Rustem Nizamov[4], Mikael Nyberg[4], Kati Miettunen[4], Niklas Nordgren[2], Jasna S. Stevanic[2], Maria Pita Guerreiro[2]*

[1]Institute of Materials, School of Engineering, EPFL, Lausanne 1015, Switzerland

[2]Bioeconomy and Health, RISE Research Institutes of Sweden, SE-114 28 Stockholm, Sweden

[3]Department of Chemistry, KTH Royal Institute of Technology, SE-100 44 Stockholm, Sweden

[4]Department of Mechanical and Materials Engineering, Faculty of Technology, University of Turku, FI-20014 Turku, Finland

Table S1. Bleaching conditions. p.c.=pulp consistency.

D					E				D				
ClO_2 %	NaOH %	p.c. %	Temp °C	Time min	NaOH %	p.c. %	Temp °C	Time min	ClO_2 %	Buffer pH	p.c. %	Temp °C	Time min
2.6	0.075	8	50	45	0.52	10	60	60	2.0	4.5	10	70	135

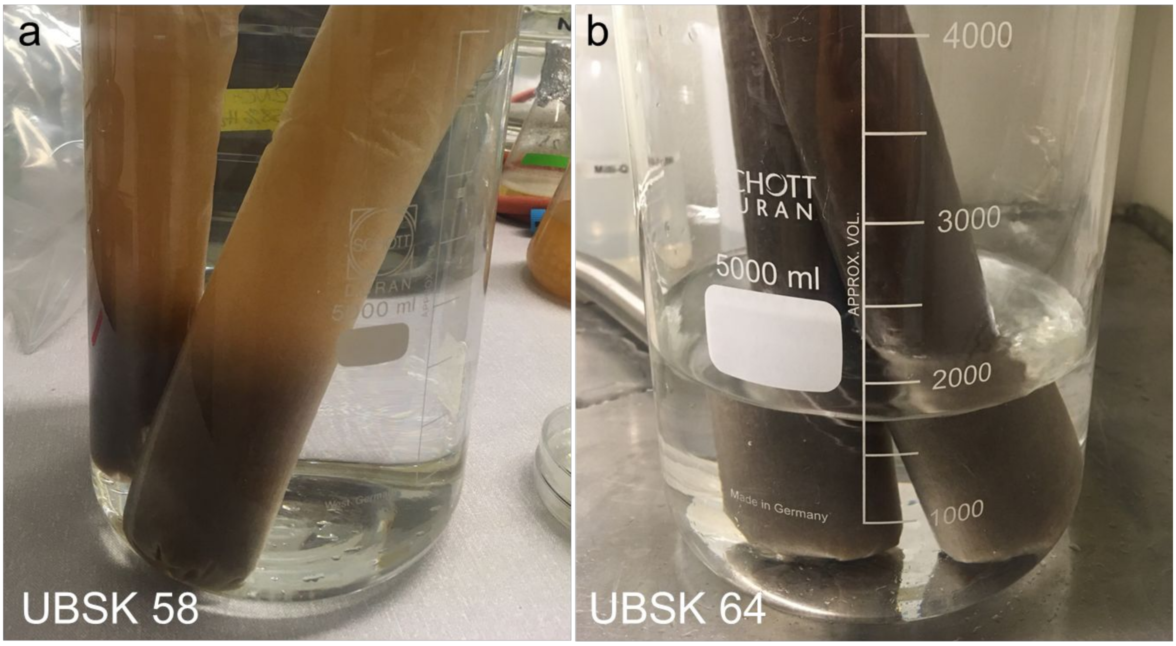

Figure S1. Snapshots of UBSK 58 (a) and UBSK 64 (b) taken in the process of changing dialysis water. UBSK 58 showed a clear sediment at this stage in the processing that disappeared after homogenization. UBSK 64 was very dark in color due to its high lignin content but uniform in consistency, with no sedimentation. BSK 58 and BSK 64 (not shown), showed similar features, without the brown color.

Table S2. Absolute carbohydrate composition including acid insoluble and acid soluble residues in mg/g of dry sample (carbohydrates as anhydrous sugars). Averages of duplicate samples.

											Relative amount in sample					
Sample	Arabinose	Galactose	Glucose	Xylose	Mannose	Acid insoluble residue	Acid soluble residue	Total lignin	Total carbo-hydrates	Total amount	Arabinose	Galactose	Glucose	Xylose	Mannose	Lignin
BSK 58	1	<1	877	28	23	7	3	10	930	940	0.14%	<0.1%	93.27%	3.00%	2.46%	1.10%
	1	<1	899	28	23	8	4	11	951	963	0.15%	<0.1%	93.36%	2.94%	2.36%	1.17%
BSK 64	1	<1	880	10	5	7	4	11	897	908	0.14%	<0.1%	96.95%	1.14%	0.54%	1.20%
	1	<1	916	11	5	10	3	13	934	947	0.15%	<0.1%	96.73%	1.14%	0.55%	1.40%
UBSK 58	1	<1	859	23	21	51	4	55	905	960	0.14%	<0.1%	89.53%	2.44%	2.16%	5.71%
	1	<1	849	23	21	56	4	60	894	954	0.14%	<0.1%	88.94%	2.41%	2.19%	6.29%
UBSK 64	1	<1	753	9	6	146	5	151	769	920	0.13%	<0.1%	81.83%	0.97%	0.62%	16.41%
	1	<1	749	9	6	150	5	155	766	921	0.13%	<0.1%	81.33%	1.00%	0.66%	16.84%
COT 64	1	<1	865	2	4	71	3	73	873	947	0.13%	<0.1%	91.38%	0.26%	0.45%	7.76%
	1	<1	894	2	4	45	3	47	902	949	0.13%	<0.1%	94.13%	0.26%	0.47%	4.98%

Table S3. Relative carbohydrate composition, % of total carbohydrate content. Averages of duplicate samples.

Sample	Arabinose	Galactose	Glucose	Xylose	Mannose	Total
BSK 58	0.1	<0,1	94.3	3.0	2.5	100
	0.1	<0,1	94.5	3.0	2.4	100
BSK 64	0.1	<0,1	98.1	1.2	0.5	100
	0.1	<0,1	98.1	1.2	0.6	100
UBSK 58	0.1	<0,1	94.9	2.6	2.3	100
	0.1	<0,1	94.9	2.6	2.3	100
UBSK 64	0.2	<0,1	97.9	1.2	0.7	100
	0.2	<0,1	97.8	1.2	0.8	100
COT 64	0.1	<0,1	99.1	0.3	0.5	100
	0.1	<0,1	99.1	0.3	0.5	100

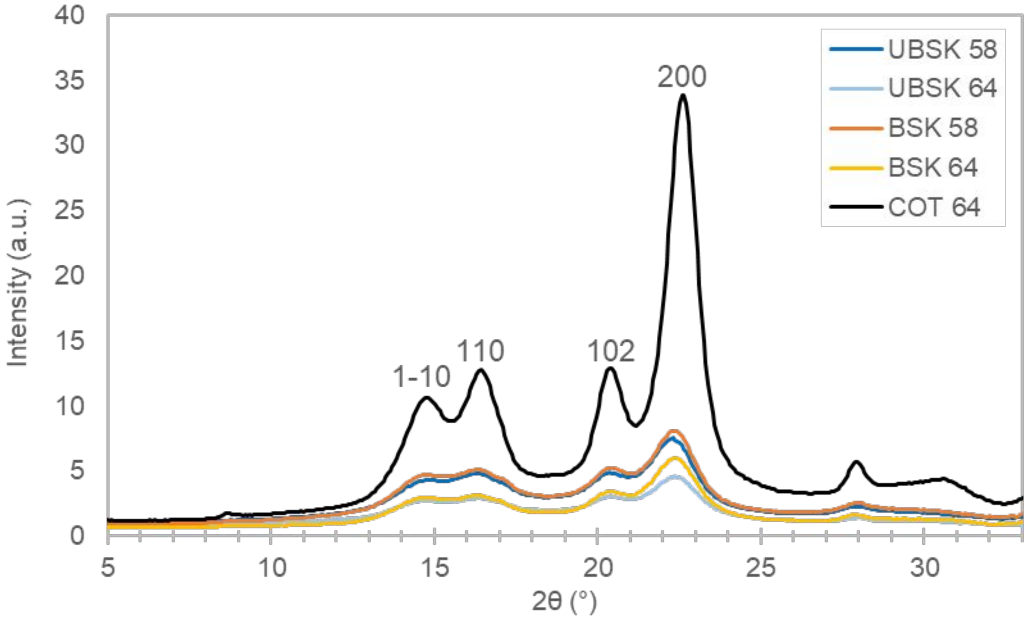

Figure S2. WAXS diffractograms of different CNCs.

Table S4. Cellulose Iβ lattice spacings from WAXS analysis with errors included in parentheses.

	Iβ d-spacings (nm)			
	(1-10)	(110)	(102)	(200)
UBSK 58	0.616 (0.003)	0.542 (0.002)	0.435 (0.0004)	0.398 (0.0001)
UBSK 64	0.607 (0.002)	0.536 (0.002)	0.435 (0.0004)	0.397 (0.0001)
BSK 58	0.604 (0.002)	0.535 (0.001)	0.434 (0.0003)	0.397 (0.001)
BSK 64	0.606 (0.001)	0.538 (0.001)	0.435 (0.0002)	0.397 (0.0001)
COT 64	0.601 (0.001)	0.538 (0.0005)	0.433 (0.0002)	0.393 (0.00005)

.

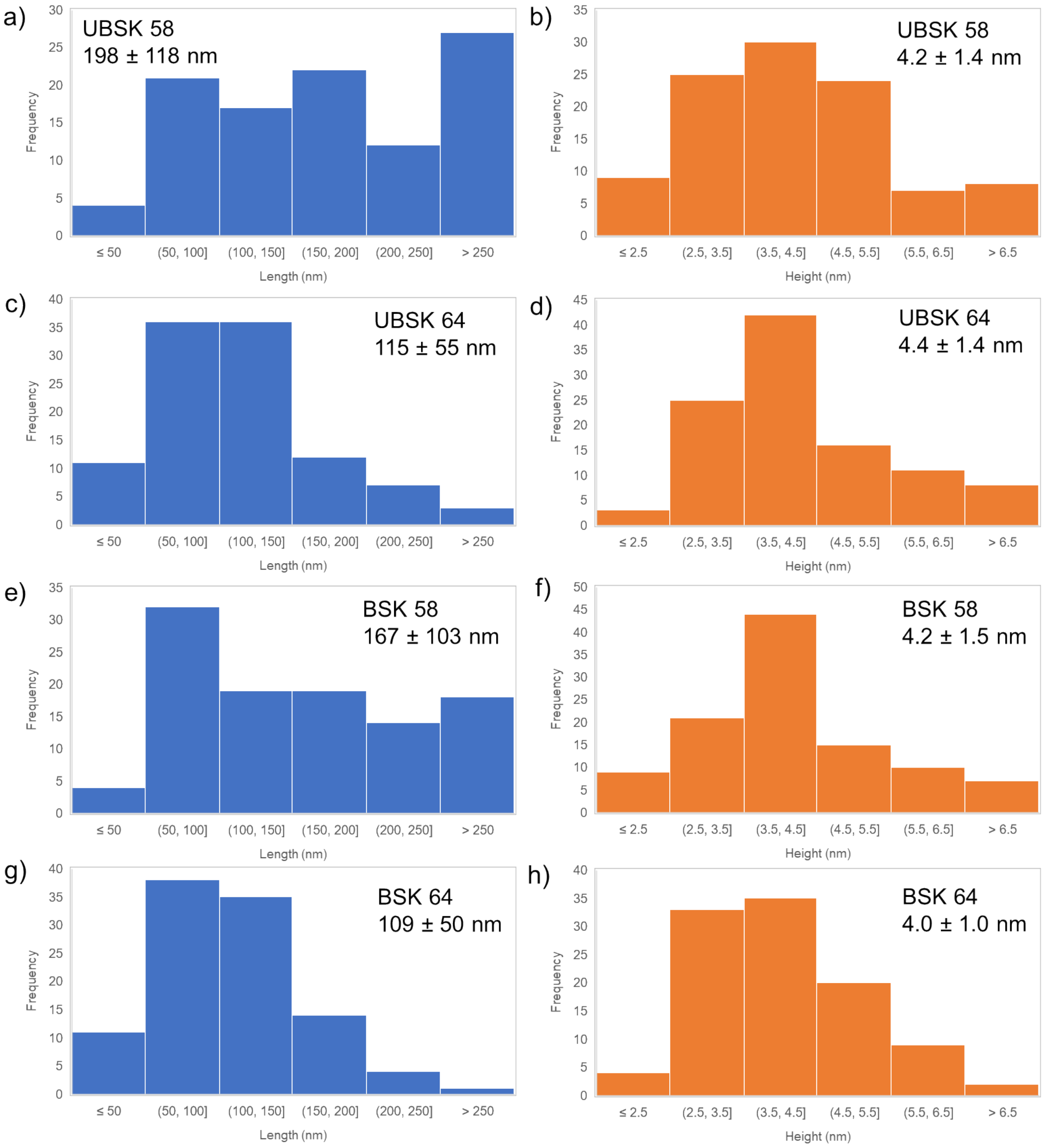

Figure S3. Size distributions of different CNCs from AFM; UBSK 58 lengths and heights, a) and b), respectively, UBSK 64 lengths and heights, c) and d), respectively, BSK 58 lengths and heights, e) and f), respectively, and BSK 64 lengths and heights, g) and h), respectively. Figure insets give mean values and standard deviations.

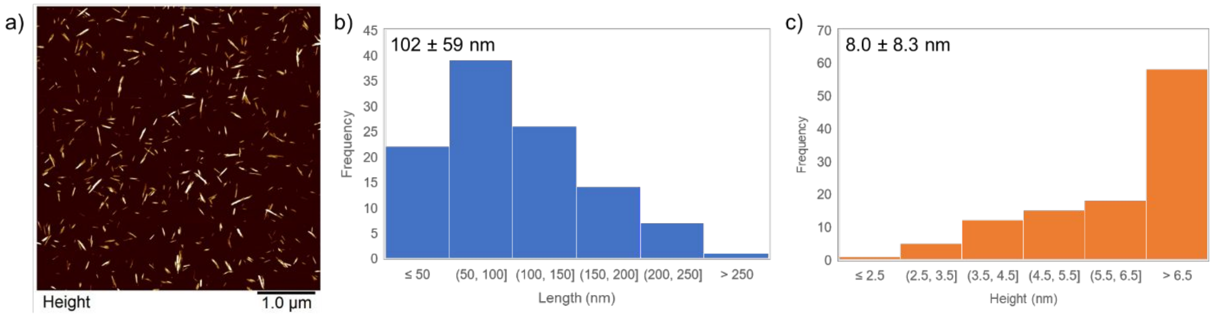

Figure S4. AFM image of COT 64 (a), with associated length (b) and height (c) distributions. Z-scale in AFM image is 16 nm.

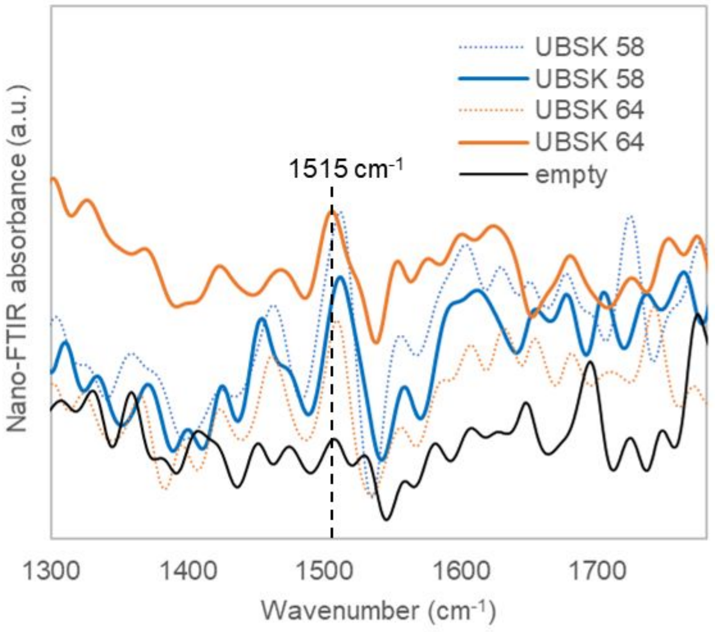

Figure S5. Typical nano-FTIR spectra acquired at random non-CNC particles/aggregates found in AFM maps of UBSK 58 (solid blue/dotted blue lines) and UBSK 64 CNCs (solid orange/dotted orange lines) spin-coated onto Si wafers and a nano-FTIR spectrum acquired from an empty area on the wafer for comparison (solid black line).

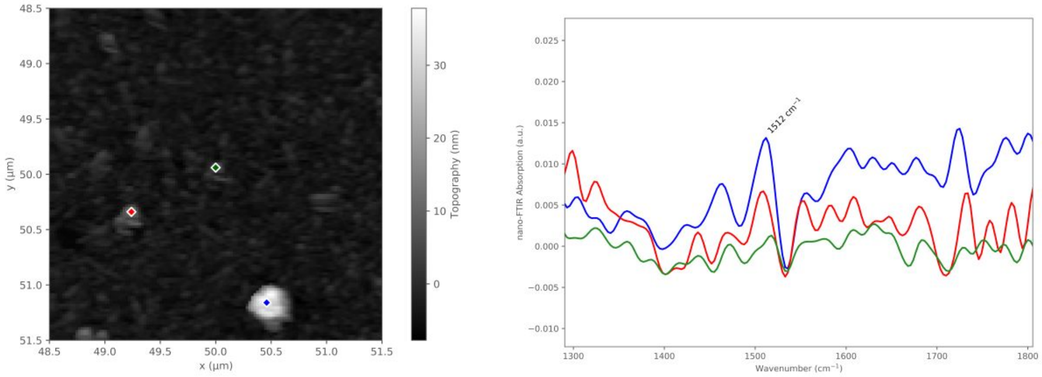

Figure S6. Typical AFM map (left) of UBSK 58 spin-coated onto a Si wafer. The color points mark the places where the nano-FTIR spectra were acquired; corresponding nano-FTIR spectra

(right). The green spectrum illustrates typical nano-FTIR spectra obtained from an area without lignin particles/aggregates.

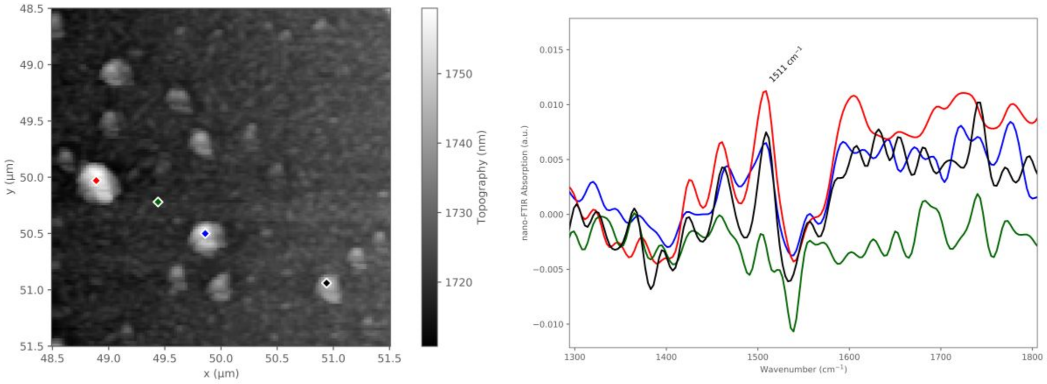

Figure S7. Typical AFM map (left) of UBSK 64 spin-coated onto a Si wafer. The color points mark the places where the nano-FTIR spectra were acquired; corresponding nano-FTIR spectra (right). The green spectrum illustrates typical nano-FTIR spectra obtained from an area without lignin particles/aggregates.

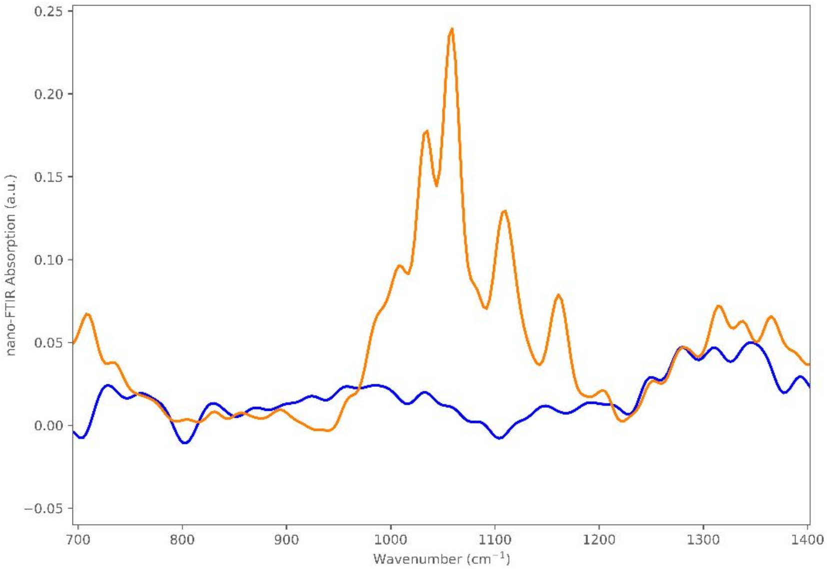

Figure S8. Representative nano-FTIR spectra acquired from lignin nanoparticles found in AFM maps of UBSK 64 CNCs spin-coated onto a Si wafer (blue) and the free-standing film of the COT 64 (orange). The latter served as a reference for a crystalline cellulose spectrum.

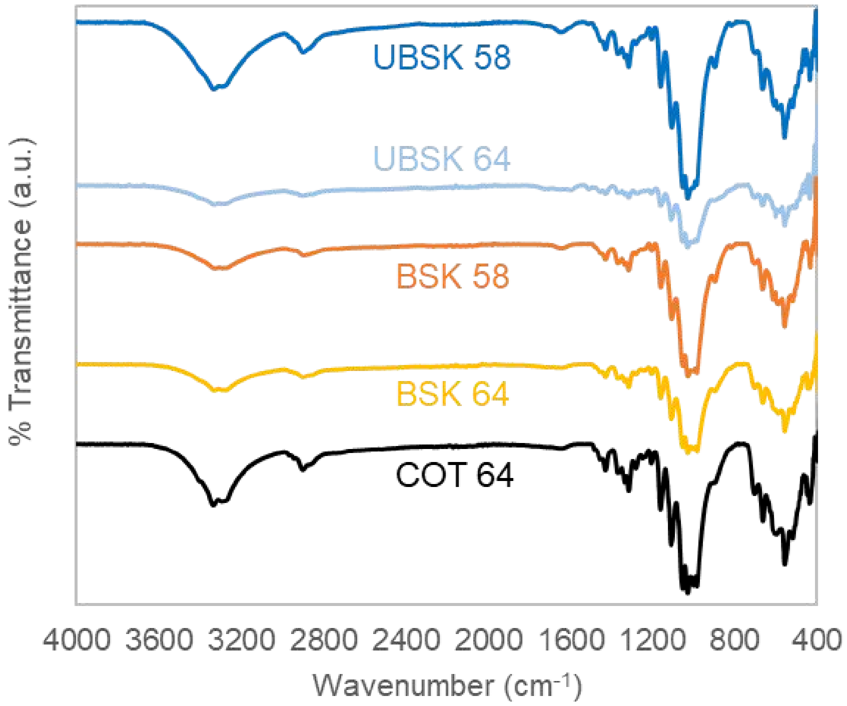

Figure S9. ATR-FTIR spectra of different CNCs showing characteristic cellulose vibrations.

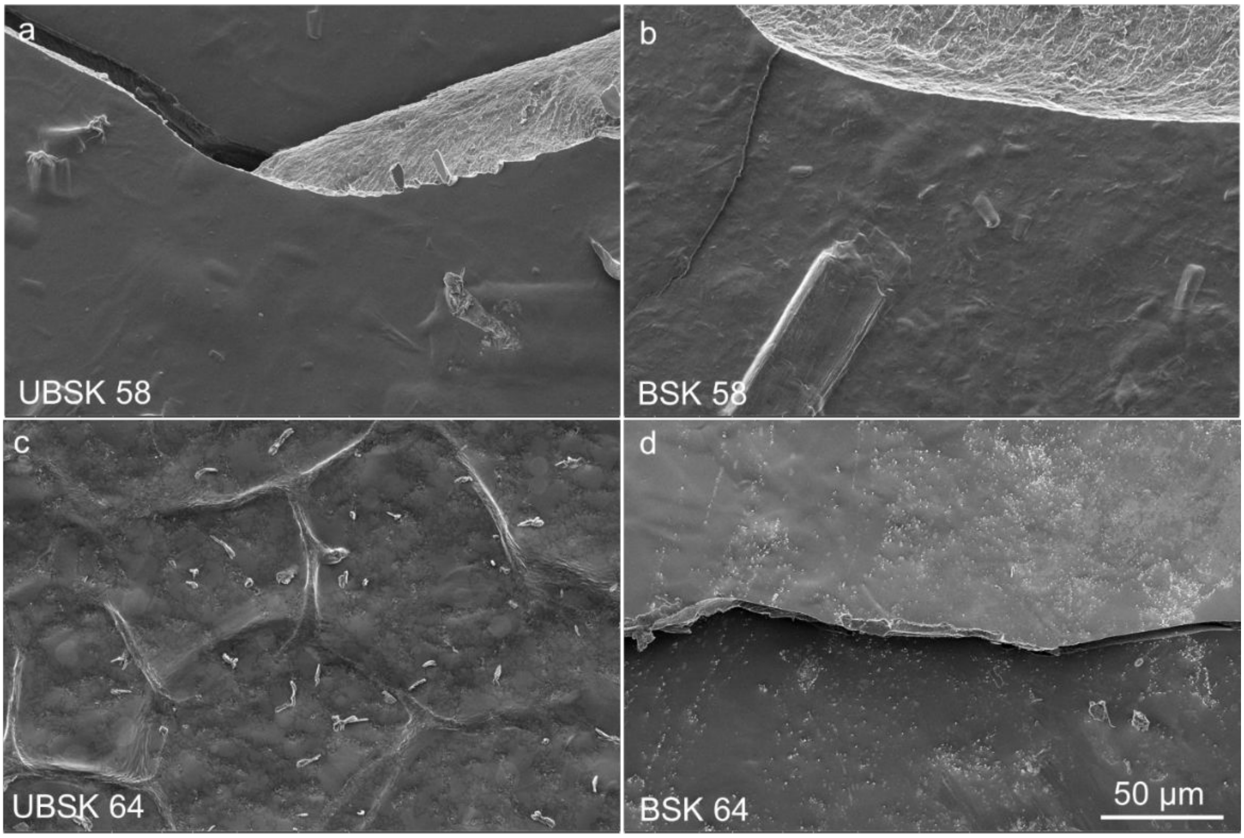

Figure S10. SEM images of CNC film surfaces; Figures a) and c) are of UBSK 58 and UBSK 64 films, respectively, and b) and d) are the BSK counterparts. The scale bars for images a-d are indicated in d).

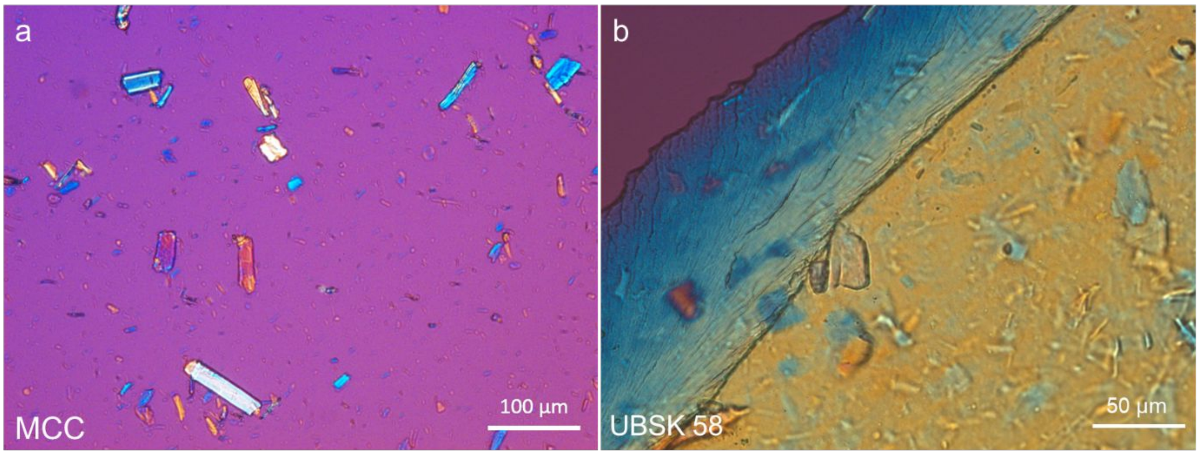

Figure S11. POM images of commercial MCC dispersion (Sigmacell, type 50) in water (0.4 wt%) showing μm-sized crystalline cellulose particles (a) and film cross-section (blue) and surface (yellow) of UBSK 58 film showing MCC distributed throughout the film (b).

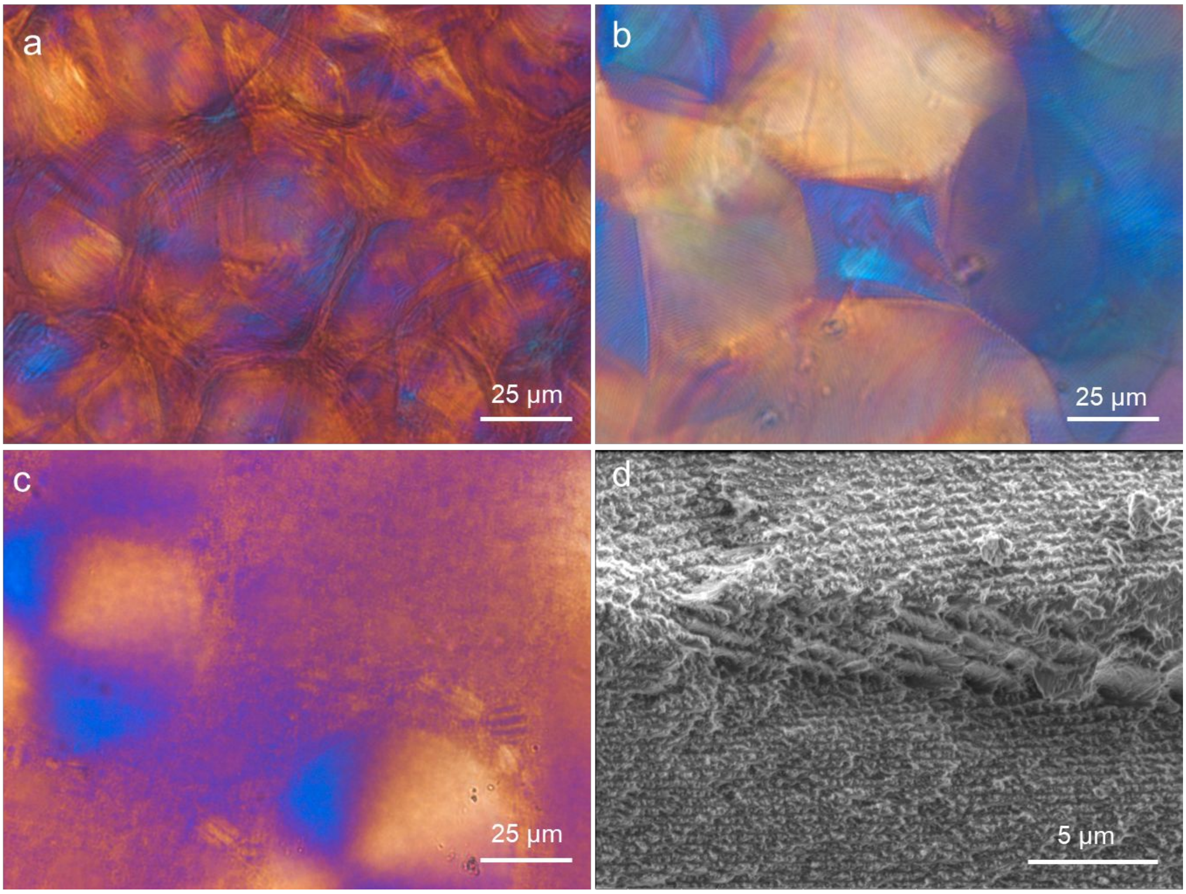

Figure S12. POM images of UBSK 64 film (a), BSK 64 film (b), and COT 64 film (c). Cross-sectional SEM of COT 64 shows tidy layered microstructure (d).

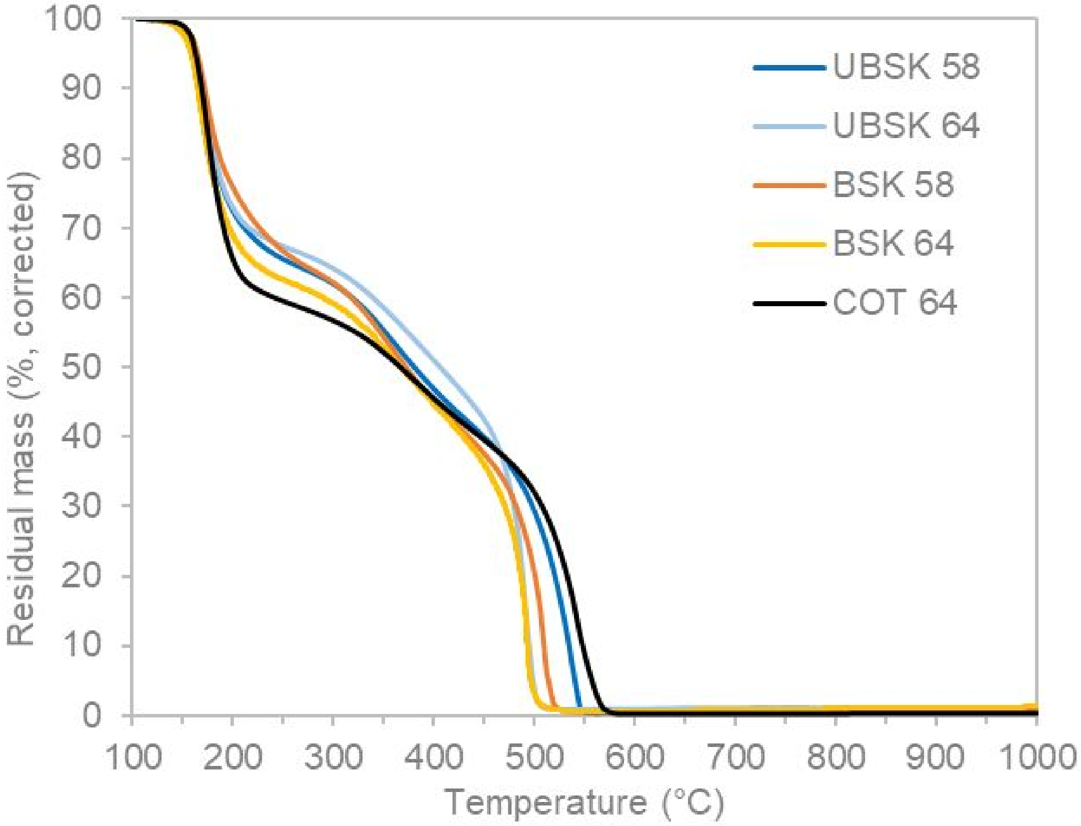

Figure S13. TGA in air of different acid-form CNC films.

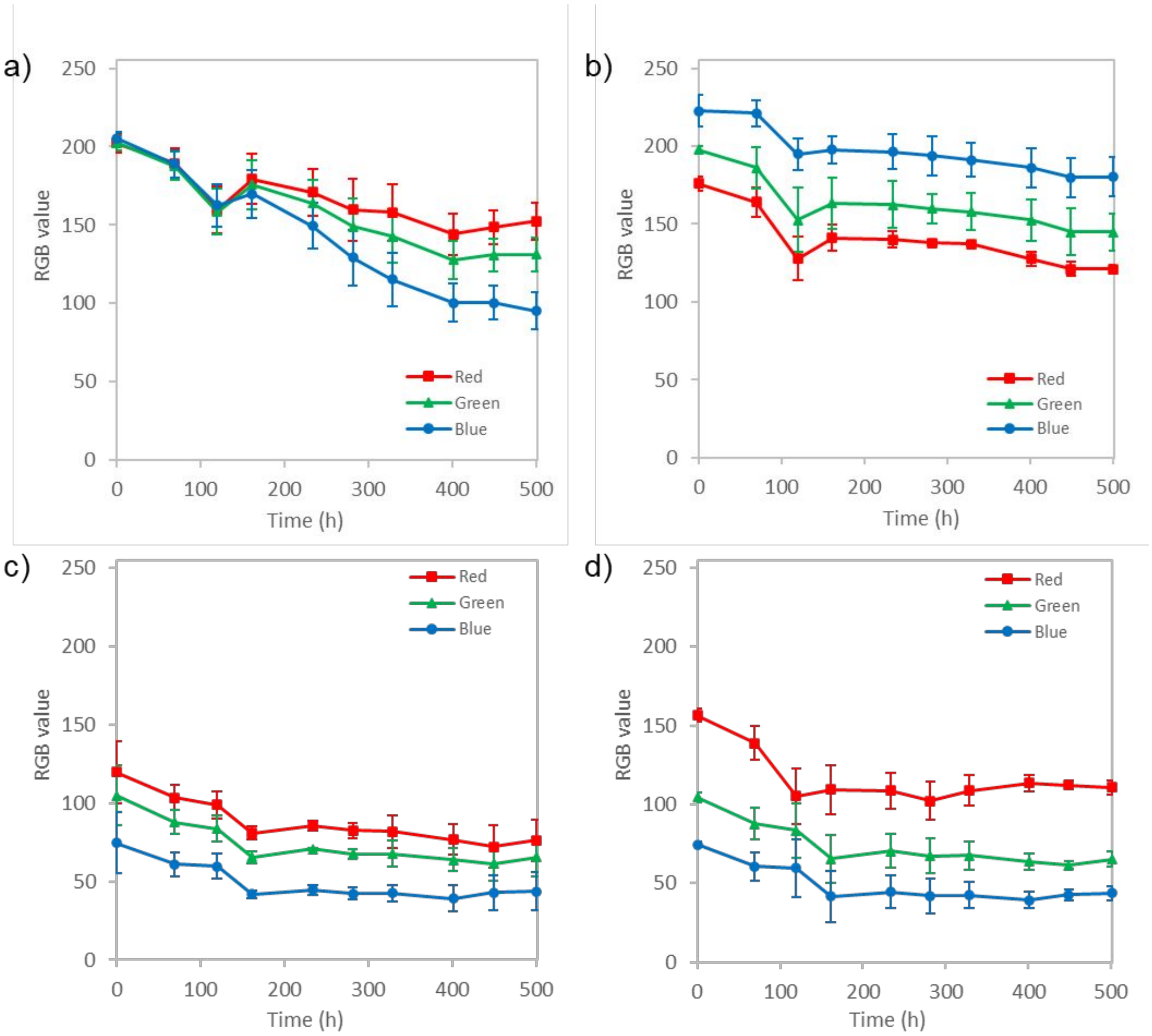

Figure S14. RGB values over time for four different CNC films: BSK 58 (a), BSK 64 (b), UBSK 58 (c), and UBSK 64 (d). Error bars indicate standard deviations between the three measurement areas.

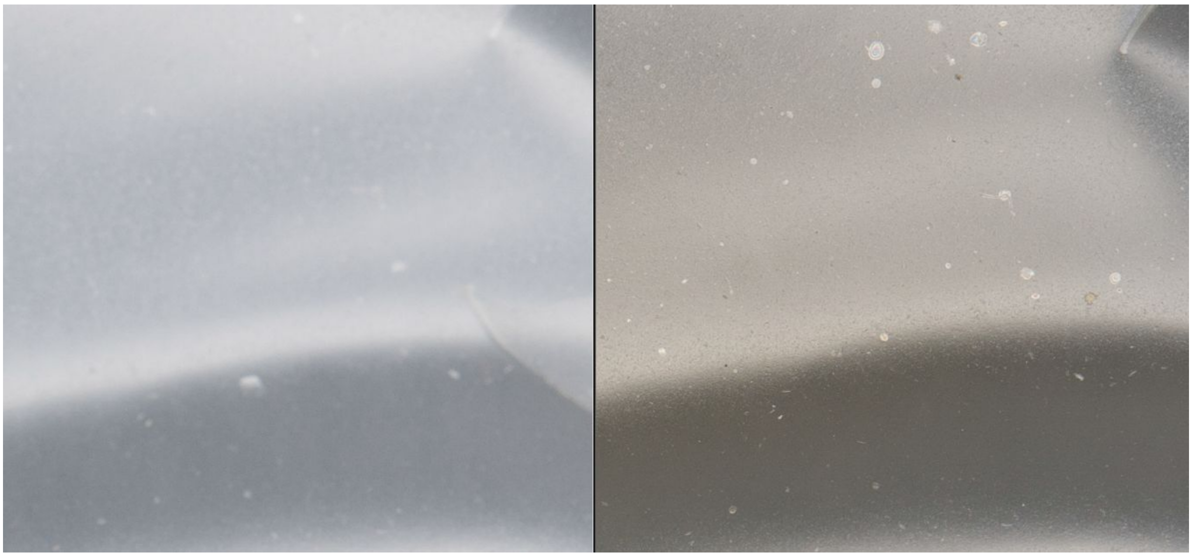

Figure S15. Initial BSK 58 film (left) and after 100 hours of exposure (right).

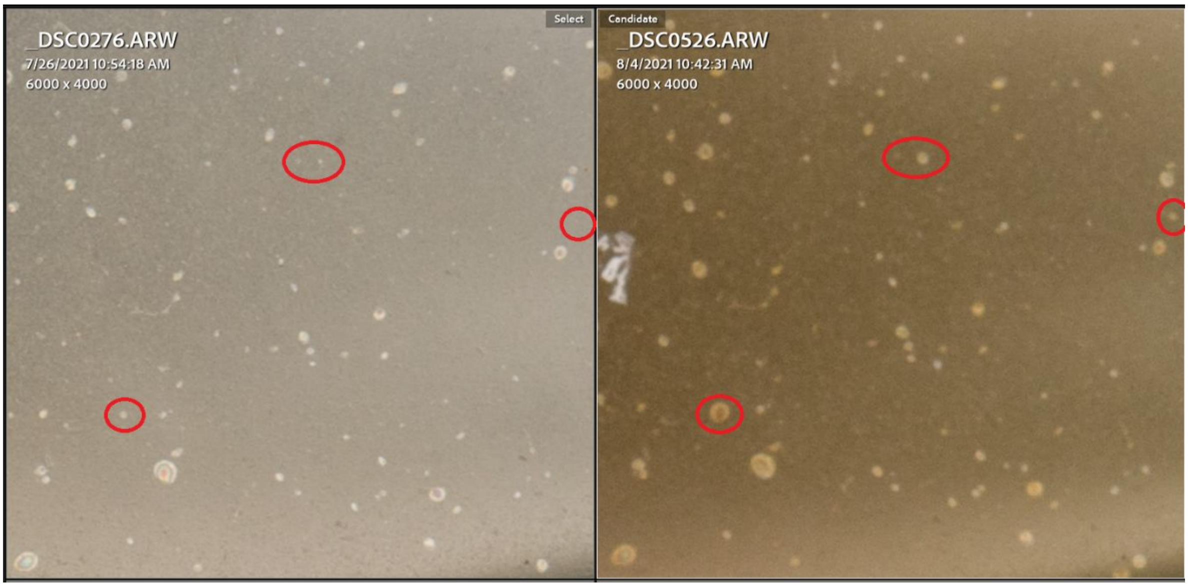

Figure S16. BSK 58 film after 200 hours of exposure (left) and after 450 hours of exposure (right).

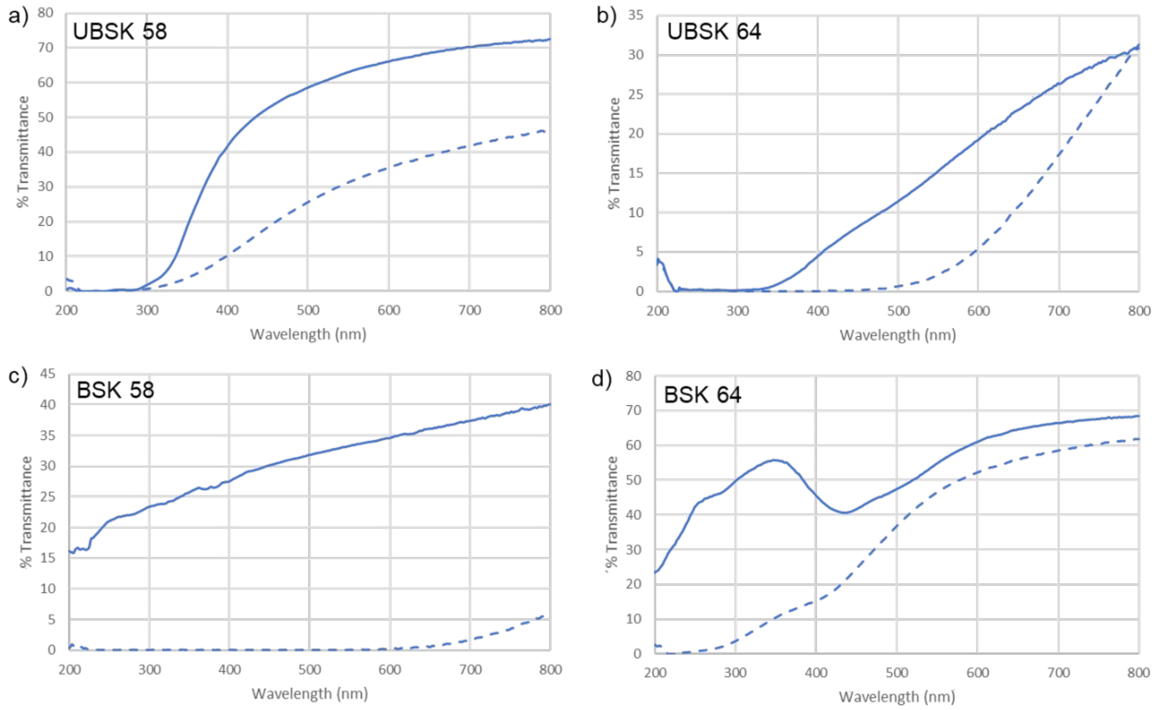

Figure S17. UV-Vis spectra before and after simulated solar exposure of UBSK 58 film (a), UBSK 64 film (b), BSK 58 film (c), and BSK 64 film (d). Spectra after exposure are indicated by dashed lines.

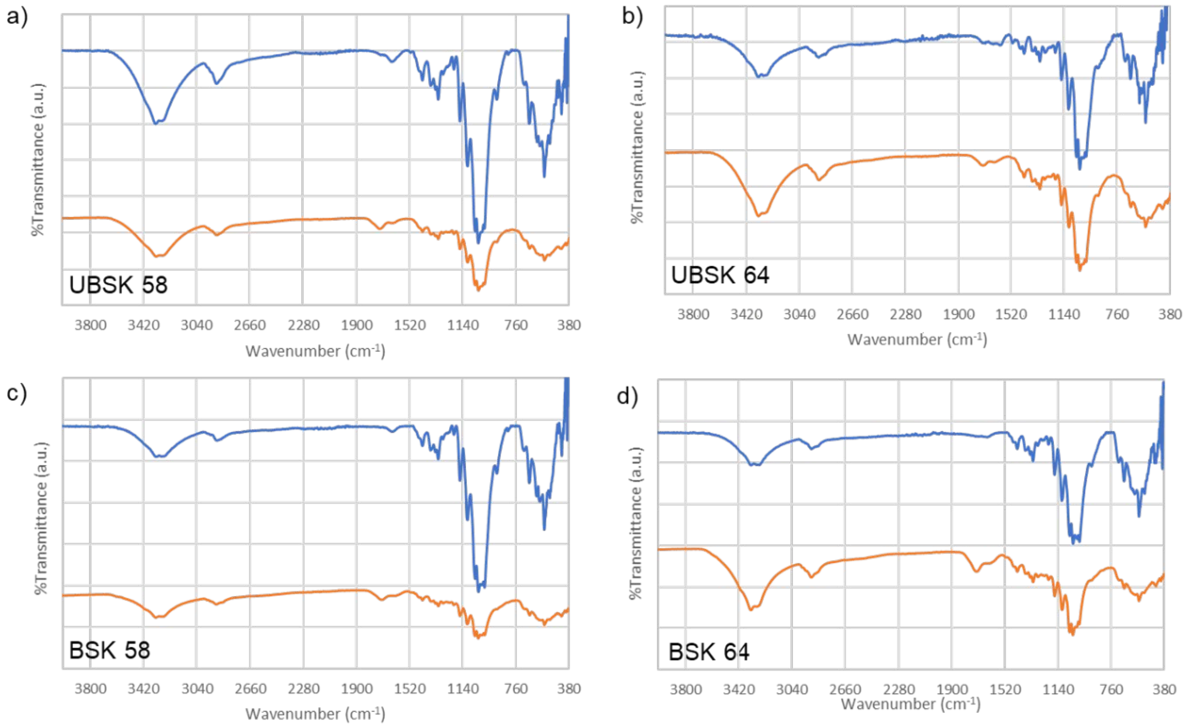

Figure S18. ATR-FTIR spectra before and after simulated solar exposure of UBSK 58 film (a), UBSK 64 film (b), BSK 58 film (c), and BSK 64 film (d). Within each graph, top spectrum is before exposure and bottom spectrum after exposure.
